# MuRF2 regulates PPARγ1 activity to protect against diabetic cardiomyopathy and enhance weight gain induced by a high fat diet

**DOI:** 10.1186/s12933-015-0252-x

**Published:** 2015-08-05

**Authors:** Jun He, Megan T Quintana, Jenyth Sullivan, Traci L Parry, Trisha J Grevengoed, Jonathan C Schisler, Joseph A Hill, Cecelia C Yates, Rudo F Mapanga, M Faadiel Essop, William E Stansfield, James R Bain, Christopher B Newgard, Michael J Muehlbauer, Yipin Han, Brian A Clarke, Monte S Willis

**Affiliations:** Department of Pathology and Laboratory Medicine, University of North Carolina, 111 Mason Farm Road, MBRB 2340B, Chapel Hill, NC USA; General Hospital of Ningxia Medical University, Yinchuan, Ningxia People’s Republic of China; Department of Surgery, University of North Carolina, Chapel Hill, NC USA; Department of Biology, University of North Carolina, Chapel Hill, NC USA; McAllister Heart Institute, University of North Carolina, 111 Mason Farm Road, MBRB 2340B, Chapel Hill, NC USA; Department of Nutrition, University of North Carolina, Chapel Hill, NC USA; Department of Pharmacology, University of North Carolina, Chapel Hill, NC USA; Department of Internal Medicine (Cardiology), University of Texas Southwestern Medical Center, Dallas, TX USA; Department of Health Promotions and Development, School of Nursing, University of Pittsburgh, Pittsburgh, PA USA; Cardio-Metabolic Research Group (CMRG), Department of Physiological Sciences, Stellenbosch University, Stellenbosch, 7600 South Africa; Sarah W. Stedman Nutrition and Metabolism Center, Duke Molecular Physiology Institute, Duke University Medical Center, Durham, NC USA; Division of Endocrinology, Metabolism, and Nutrition, Department of Medicine, Duke University Medical Center, Durham, NC USA; East Chapel Hill High School, Chapel Hill, NC USA; Novartis, Novartis Institutes for BioMedical Research, Inc., 400 Technology Square, Boston, MA 601-4214 USA

**Keywords:** MuRF2, Diabetic cardiomyopathy, Post-translational modification, Multi-ubiquitin, PPAR, Ubiquitin ligase

## Abstract

**Background:**

In diabetes mellitus the morbidity and mortality of cardiovascular disease is increased and represents an important independent mechanism by which heart disease is exacerbated. The pathogenesis of diabetic cardiomyopathy involves the enhanced activation of PPAR transcription factors, including PPARα, and to a lesser degree PPARβ and PPARγ1. How these transcription factors are regulated in the heart is largely unknown. Recent studies have described post-translational ubiquitination of PPARs as ways in which PPAR activity is inhibited in cancer. However, specific mechanisms in the heart have not previously been described. Recent studies have implicated the muscle-specific ubiquitin ligase muscle ring finger-2 (MuRF2) in inhibiting the nuclear transcription factor SRF. Initial studies of MuRF2−/− hearts revealed enhanced PPAR activity, leading to the hypothesis that MuRF2 regulates PPAR activity by post-translational ubiquitination.

**Methods:**

MuRF2−/− mice were challenged with a 26-week 60% fat diet designed to simulate obesity-mediated insulin resistance and diabetic cardiomyopathy. Mice were followed by conscious echocardiography, blood glucose, tissue triglyceride, glycogen levels, immunoblot analysis of intracellular signaling, heart and skeletal muscle morphometrics, and PPARα, PPARβ, and PPARγ1-regulated mRNA expression.

**Results:**

MuRF2 protein levels increase ~20% during the development of diabetic cardiomyopathy induced by high fat diet. Compared to littermate wildtype hearts, MuRF2−/− hearts exhibit an exaggerated diabetic cardiomyopathy, characterized by an early onset systolic dysfunction, larger left ventricular mass, and higher heart weight. MuRF2−/− hearts had significantly increased PPARα- and PPARγ1-regulated gene expression by RT-qPCR, consistent with MuRF2’s regulation of these transcription factors in vivo. Mechanistically, MuRF2 mono-ubiquitinated PPARα and PPARγ1 in vitro, consistent with its non-degradatory role in diabetic cardiomyopathy. However, increasing MuRF2:PPARγ1 (>5:1) beyond physiological levels drove poly-ubiquitin-mediated degradation of PPARγ1 in vitro, indicating large MuRF2 increases may lead to PPAR degradation if found in other disease states.

**Conclusions:**

Mutations in MuRF2 have been described to contribute to the severity of familial hypertrophic cardiomyopathy. The present study suggests that the lack of MuRF2, as found in these patients, can result in an exaggerated diabetic cardiomyopathy. These studies also identify MuRF2 as the first ubiquitin ligase to regulate cardiac PPARα and PPARγ1 activities in vivo via post-translational modification without degradation.

**Electronic supplementary material:**

The online version of this article (doi:10.1186/s12933-015-0252-x) contains supplementary material, which is available to authorized users.

## Background

The leading cause of morbidity and mortality worldwide is cardiovascular disease [1], frequently accompanied by the dysregulation of fatty acid metabolism associated with diabetes mellitus (DM). In the presence of DM, the morbidity and mortality of cardiovascular disease is increased and represents an important independent mechanism by which heart disease is exacerbated [2, 3]. The characteristic disturbances in myocardial energy and fatty acid homeostasis found in DM are mediated primarily by a network of peroxisome proliferator-activated receptor (PPAR) transcription factors that direct the energy substrates and determine the myocardial homeostasis [4, 5]. Chronic activation of PPARs in DM leads to an increase in free fatty acid uptake/oxidation corresponding to the level of insulin resistance in cardiomyocytes [6]. The increased reliance on fatty acid metabolism decreases the efficiency of the heart by increasing the amount of oxygen needed to create the needed energy, resulting in lipotoxicity [7]. The ligand (fatty acid)-driven activation of PPAR transcription factors regulate the expression of target genes, which control the uptake, utilization, oxidation, and storage of fatty acids [8]. In the heart, all three PPAR receptors have been identified (PPARα, PPARδ/β, and PPARγ) and implicated in cardiovascular disease [9].

Insulin resistance is a risk factor for left ventricular (LV) dysfunction and heart failure and is one of the hallmarks of type 2 DM [10]. Despite hyperinsulinemia and hyperglycemia, the diabetic heart relies almost exclusively on fatty acid utilization [11] in both rodent models and humans with excessive fat intake [12]. The resulting increase in fatty acid increases reaction oxygen species (ROS) production and accumulation of lipid intermediates [e.g. diacylglycerol (DAG)], which have a profound impact on insulin signaling [13]. The c-JUN NH 2-terminal kinase (JNK) and inhibitor κB kinase (IKK), activated by ROS [14, 15], parallel activation of protein kinase C (PKC) by DAG, all of which act to down-regulate insulin action by preventing insulin receptor substrate-1 (IRS-1) phosphorylation [13]. High systemic fatty acid uptake also inhibits Akt signaling, resulting in the downregulation of forkhead box O (FOXO) transcription factors [16, 17], while the increased ROS activate nuclear factor kappa B (NF-κB) [18], both of which contribute to the development of cardiac hypertrophy [12, 19].

The muscle ring finger (MuRF) family of ubiquitin ligases, including MuRF2 (*Trim55*), was identified in 2001 as a highly homologous group of proteins that homo- and hetero-dimerize through their coiled-coil domains [20]. This family of proteins is found in striated muscle, including skeletal and cardiac myocytes and was originally found to be a critical regulator of microtubule assembly during models of skeletal muscle development [21, 22]. Recent studies have detailed the importance of MuRF2 in the earliest stages of skeletal muscle differentiation and myofibrillogenesis in vivo [23]. In the present study, we identify that endogenous cardiomyocyte MuRF2 inhibits multiple PPAR isoforms, primarily PPARγ (but to a lesser extent PPARδ/β and PPARα). Given the relative importance of PPARs in the development of diabetic cardiomyopathy and the downstream pathophysiology, we challenged MuRF2−/− mice to a 60% fat diet-induced cardiomyopathy recently described [24, 25]. With PPAR signaling at the center of regulating fatty acid oxidation and mediating the pathogenesis of type 2 DM induced cardiomyopathy, we hypothesized that if MuRF2−/− hearts had enhanced PPAR signaling, they would undergo an accelerated cardiomyopathy due to MuRF2’s direct regulation of PPAR activity. We identified that MuRF2−/− hearts undergo an exaggerated diabetic cardiomyopathy, resulting from MuRF2’s multi-ubiquitination of PPARα and PPARγ1 in a proteasome-independent (non-degradatory) mechanism. These studies identify the first ubiquitin ligase to regulate PPAR via post-translational ubiquitination.

## Methods

### Animals and high fat diet-induced diabetic cardiomyopathy model

All experiments described used age-matched mice or littermates, male and female. All experiments were approved by the Institutional Animal Care and Use Committee (IACUC) review boards at the University of North Carolina and were performed in accordance with federal guidelines. Ten week-old MuRF2−/− and strain-matched wild type mice [26] were fed a high fat diet (60% fat, 20% protein, and 20% carbohydrates) for 26 weeks as previously described [24]. Baseline body weight, blood glucose, serum insulin, serum triglyceride, and total cholesterol levels along with cardiac function were obtained prior to starting the diet. Body weight, blood glucose, and serum insulin levels measured every 2 weeks; echocardiography was performed every 3 weeks. An MRI was performed at baseline, 6, 12, and 22 weeks to detect body composition changes. After 26 weeks, mice were anesthetized with isoflurane, euthanized with cervical spine dislocation, and heart, liver, gastrocnemius, soleus, and tibialis anterior muscles were collected in cryovials, flash frozen, and stored at −80°C.

### Mouse echocardiography

Conscious transthoracic echocardiography was performed on mice at the indicated time points using a VisualSonics Vevo 2100 ultrasound biomicroscopy system (VisualSonics, Inc., Toronto, Ontario, Canada). Investigators were blinded to mouse genotype. Two-dimensional M-mode echocardiography was performed in the parasternal long-axis view at the level of the papillary muscle on loosely restrained mice. Anterior and posterior wall thickness was measured as distance from epicardial to endocardial leading edges. Left ventricular internal diameters were also measured. Left ventricular systolic function was assessed by ejection fraction (LV EF% = [(LV Vol; d-LV Vol; s/LV Vol; d) × 100] and fractional shortening (%FS = [(LVEDD − LVESD)/LVEDD] × 100). Measurements represent the average of three cardiac cycles from each mouse.

### Body composition measurement

Conscious low-resolution nuclear magnetic resonance imaging was used to measure body composition of each mouse at baseline, 6, 12, and 22 weeks using an EchoMRI 3-in-1 Body Composition Analyzer for Live Small Animals (Mice) (EchoMRI, LLC, Houston, TX, USA) [27]. Body fat and lean body mass was then calculated as a proportion of total body weight collected just prior to analysis as previously described [28].

### Blood collection, serum separation, and methods for glucose, insulin, triglyceride, and total cholesterol measurements

After overnight fast, ~200 µl whole blood was collected by submandibular vein lancet bleed (glucose) or brachial sinus puncture (remaining assays). One microliter whole blood was analyzed via glucometer (PrecisionXtra, Abbott Diabetes Care Inc., Alameda, CA, USA) and test strip (Abbott Diabetes Care Ltd., Witney, Oxon, UK). Blood collected in serum separator tubes for the remaining tests was incubated on ice for 90 min, and centrifuged at 1,600×*g* (20 min at 4°C). Insulin levels were measured using the Insulin Enzyme Immunoassay Kit (Cayman Chemical, Cat. #589501, Ann Arbor, MI 48108, USA) according to the manufacturer’s instructions as previously described [29]. Serum triglyceride and cholesterol levels were measured using an automated chemical analyzer (Vitro 350, OrthoClinical Diagnostics Company, Rochester, NY, USA).

### Fatty acid extraction and triglyceride assay

Fatty acid extraction and tissue triglyceride concentrations were determined on flash frozen heart tissue, liver tissue, and skeletal tissue as previously described [30]. Briefly, 25–50 mg of heart, liver and skeletal muscle was homogenized 15–30 s with a bladed homogenizer (Power Gen 125, Cat. #14-261, setting 6, Fisher Scientific, Inc., Pittsburgh, PA, USA) in 10× (v/w) ice cold lysis buffer [20 mM Tris base, 1% Triton-X100, 50 mM NaCl, 250 mM NaF, 5 mM Na4P2O7-10H2O, 1 tablet protease inhibitor (Roche Inc., Cat. #11836153)] and incubated at 4°C for 1 h. Two hundred microliters of homogenate was transferred to chloroform resistant tubes, mixed with 0.4 ml methanol and 0.8 ml chloroform, placed on the rocker at 4°C for at least 30 min. Potassium chloride (0.24 ml 0.88% KCl) was added, samples vortexed, and centrifuged at 1,000×*g* for 15 min at 4°C. The bottom layer of CHCl_3_ was then transferred and this process was repeated with another 0.8 ml of chloroform and the combined CHCl_3_ layers were then dried under N_2_. One hundred microliters of a tert-butanol:methanol:Triton X-100 solution (3:1:1, v/v/v) was added to each tube and samples were stored at −20°C. Glycerol standard 2.5 mg/dl (Sigma, Inc., Cat. #G1394), free glycerol reagent (Sigma Aldrich, Inc., Cat. #F6428) and triglyceride reagent (Sigma Aldrich, Inc., Cat. #T2449) were used to measure triglyceride concentrations. Five microliters of the samples were added to a 96-well plate. Working reagent was added to the samples (four volumes of free glycerol reagent: 1 volume of triglyceride reagent). This was left to incubate, rocking, at room temperature for 15 min. Then absorbance was measured per sample at 540 nm using the Clariostar High Performance Multimode Microplate Reader (BMG LABTECH, San Francisco, CA, USA) and normalized to tissue weight.

### Tissue glycogen assay (acid hydrolysis method)

Tissue glycogen was measured from heart, liver and skeletal muscle using a colorimetric tissue glycogen assay kit (Sigma, Inc., Cat. #G3293) as previously described [31]. Briefly, 15–25 mg of tissue was powdered in liquid nitrogen, collected in a pre-chilled 2 ml tube, 0.5 ml 1 N HCl added, then homogenized with bladed homogenizer (Fisher Scientific, Power Gen 125, Cat. #14-261, setting 6, Pittsburgh, PA, USA) under a hood. The resulting homogenate (100 µl) was quickly added to 100 µl 1 N NaOH and kept on ice until heated in HCl at 95°C for 90 min, mixing every 30 min, cooled to RT and 0.4 ml 1 N Na OH was added to neutralize the sample. After the sample was centrifuged at 14,000×*g* for 10 min at RT, the supernatant was used for glucose analysis using a hexokinase-dependent assay kit (Sigma, Inc., Cat. #G3293) according to the manufacturer’s instructions. Briefly, 10 μl (liver) or 20 μl (heart and gastrocnemius) of supernatant was put into a 96-well plate, mixed with 200 μl of reagent, incubated at room temperature for 15 min, and the absorbance was measured at 340 nm.

### Cell culture

Cos-7 and HEK293 cells were cultured in DMEM containing 10% FBS, 100 unit/ml penicillin and 0.1% mg/ml streptomycin. HL-1 cardiomyocytes were cultured in supplemented Claycomb medium containing 10% FBS, 100 unit/ml penicillin, 0.1% mg/ml streptomycin, 0.1 mM norepinephrine and 2 mM l-glutamine. All cells were incubated at 37°C in a 5% CO_2_ humidified atmosphere.

### Confocal microscopy

HL-1 cardiomyocytes (2.5 × 10^5^/well/50% confluent) plated on Gelatin/Fibronectin were co-transfected with Flag-PPARγ1 and HA-MuRF2 using Lipofectamine LTX & PLUS (Invitrogen, lot#1397274) according to the manufacturer’s instructions. The ratios of LTX/DNA and PLUS/DNA (μl/μg) both were 2:1. Equal amounts of DNA were transfected by adjusting with empty vectors. After 48 h of transfection, the cells were fixed with 4% paraformaldehyde and blocked in 5% goat serum with 0.2% TritonX-100 at room temperature for 1 h. Cells were incubated with Rb anti-Flag (Sigma F7425, 1:100, 4°C, overnight or Ms anti-HA (Sigma H9658, 1:100, 4°C, overnight). Cells were washed and incubated with anti-Ms 488 to detect HA-MuRF2 (Invitrogen, 1:1,000) or anti Rb 568 (Invitrogen, 1:1,000) for 1 h at room temperature. The membranes were cut into 1 × 1 cm sections and mounted to glass slides with Fluoro-Gel Anti-fade mounting medium with DAPI (EMS, Hatfield, PA Cat. #17983-20) and analyzed by fluorescent confocal microscopy using a Zeiss CLSM 710 Spectral Confocal Laser Scanning Microscope.

### RNA isolation and quantitative PCR analysis of PPAR-regulated gene expression

Total RNA was isolated using TRIzol reagent according to the manufacturer’s protocols (Life Technologies, Inc., Cat. #15596-026). Approximately 25 mg of cardiac ventricular tissue was put into TRIzol reagent and homogenized on ice (Fisher Scientific, Power Gen 125, setting 5). Total mRNA expression was determined using a two-step reaction. cDNA was made from total RNA using the iScript™ Reverse Transcription Supermix for RT-qPCR kit (Cat. #170-8841, BIO-RAD), with a total volume of 20 µl per reaction. The complete reaction mix was incubated in an Eppendorf Cycler (Hamburg, Germany) using the following protocol: priming 5 min at 25°C, reverse transcription 30 min at 42°C, RT inactivation 5 min at 85°C. PCR products were amplified on a Roche Lightcycler 480II system using cDNA, Taqman Probes (Applied Biosciences™), and Lightcycler 480 Probe Master Mix 2X (Cat. #04 707 494 001). The TaqMan probes used in this study are Mm00430615_m1 (ACC1), Mm00443579_m1 (ACOX1), Mm00475794_m1 (ADRP), Mm00599660_m1 (LCAD), Mm00431611_m1 (MCAD), Mm00440939_m1 (PPARα), Mm01305434_m1 (PPARβ), Mm00443325_m1 (PDK4), Mm00487200_m1 (CPT1b), Mm00441480_m1 (Glut1, Slc2a1), Mm01245502_m1 (Glut4, Slc2a4), Mm01309576_m1 (PFK), Mm00432403_m1 (CD36, FAT), Mm01185221_m1 (MuRF1, Trim63), and Mm01292963_g1 (MuRF2, Trim55), Hs99999901_s1 (18S), Mm00440359_m1 (α-MHC, Myh6), Mm00600555_m1 (β-MHC, Myh7), Mm01255747_g1 (ANP), Mm00435304_g1 (BNP), Mm00808218_g1 (SK α-actin) (Applied Biosystems, Inc., Foster City, CA, USA). Assay of PPARγ1 was performed using the Roche Universal Probe technology, including forward primer (gggctgaggagaagtcacac) and reverse primer (gggctgaggagaagtcacac) in conjunction with UPL probe #92 (Roche, Inc., Cat. #04692098001). Samples were run in triplicate and relative mRNA expression was determined using 18S as an internal endogenous control. RNase-free water, 2× Master Mix, Taqman Probe or Roche UPL primer and probe, and cDNA were used for each reaction.

### Western blot

Western analysis of ventricular tissue was performed on lysates created from ~25 mg tissue placed in 1× Cell Signaling Lysis Buffer (for 10 ml: 1 ml 10× Cell Signaling Lysis Buffer, Cat. #9803S; 0.108 g β-glycerol phosphate, Sigma, Cat. #G6251; 1 tablet protease inhibitor, Roche Cat. #11 836 153 001; 100 μl 100X phosphatase inhibitor cocktail, Roche Cat. #04 906 837 001) and was homogenized on ice (Fisher Scientific, Power Gen 125, setting 5) for ~15–20 s. The homogenate was incubated on ice for 30 min, centrifuged at 4°C, ×16,000×*g* for 15 min and the supernatant stored at −80°C. Protein concentration was determined using the Bio-Rad DC Protein Assay Reagent Package (Bio-Rad Laboratories, Inc., Hercules, CA, Cat. #500-0116). Proteins (30–50 μg/lane) were resolved on NuPAGE Bis–Tris or Tris–Acetate 10 well gels. Mouse anti-NFκB p65, rabbit anti-phospho-NFκB p65 (Ser536), rabbit anti-phospho-NFκB p65 (Ser468) were used to measure NFκB signaling (Cell Signaling Technologies, Cat. #6956, #3033, and #3039, 1:500). IRS-1 signaling was detected using rabbit anti-phospho-IRS-1 (Ser1101) and rabbit anti-IRS-1 (Cell Signaling Technologies, Inc. Cat. #2385 and #2383, 1:500). cJun signaling was detected by rabbit anti-p-cJun (Ser73), Rb anti-p-cJun (Thr91) or Rb anti-cJun 60A8 (Cell Signaling Technologies, Cat. #9164, #2303, #9165, 1:500). Rabbit anti-PPARα (Abcam Inc. Cat. #24509,1:1000), rabbit anti-PPARβ/δ (Abcam Inc. Cat. #8937, 1:500), and rabbit anti-PPARγ (Cell Signaling Technologies, Inc. Cat. #2443, 1:500) were used to measure protein expression of the PPAR isoforms. MuRF2 protein expression was detected by goat anti-MuRF2 (Abcam Inc. Cat. #4387, 1:1000). Primary antibodies were diluted in 5% milk or bovine serum albumin and incubated at 4°C overnight. HRP-labeled secondary antibodies against mouse (Sigma #A9917, 1:10,000), goat (Sigma #A4174, 1:10,000), and rabbit (Sigma #A9169, 1:5,000) were used to detect the primary antibodies diluted in 1× TBS-T and incubated 1 h at room temperature. Mouse anti-β-actin (Sigma, Inc., Cat. #A2228, 1:4,000) and mouse anti-GAPDH (Sigma, Inc., Cat. #G8795, 1:4,000) were used as a loading controls throughout. Secondary antibody HRP was detected using ECL Select (GE Healthcare, Cat. #RPN2235) and imaged using the MultiDoc-it Imaging System (UVP, LLC Ultra-violet Products, Ltd., Upland, CA, USA).

### Immunoprecipitation studies

HEK293 cells were cotransfected with p3XFlag-PPARγ1 and pcDNA3.1-HA-MuRF2 or pcDNA3.1-HA-MuRF2ΔRing DNA plasmids. After 28 h of transfection, cells were lysed using RIPA buffer (Sigma, Inc., Cat. #R0278). Protein concentration was determined using Bio-Rad DC Protein Assay. 60 μl EZview Red Anti-Flag M2 Affinity Gel beads (Sigma, Inc., Cat. #F2426) were washed twice using 1× TBS, after the addition of 250 μg protein lysates, samples were gently agitated on a roller shaker overnight at 4°C. After three washes with 1xTBS, the proteins were eluted by 30 μl of 2× LDS Sample Buffer (NuPAGE LDS Sample Buffer, Lot#1452697) and boiled for 5 min at 100°C. Samples were analyzed by immunoblotting.

### Total O-GlcNAc expression

Total *O*-GlcNAc expression was determined by SDS-PAGE as previously described [32], using anti-*O*-GlcNAc (RL-2, Santa Cruz Biotechnology, Santa Cruz CA) on PVDF blocked with 1% bovine serum albumin dissolved in TBS-T solution for 20 min, followed by an overnight incubation with *O*-GlcNAc antibody (1:1,000) at 4°C. Secondary antibody (goat-anti-mouse IgG-HRP, Santa Cruz Biotechnologies, Santa Cruz CA; 1:4,000) incubated for 1 h at room temperature, washed with TBS-T, then visualized with enhanced chemiluminescence (ECL) on the ChemiDoc™ XRS+ system with Image Lab™ Software v2.0 (Bio-Rad Laboratories, Hercules CA, USA). Total *O*-GlcNAcylation (per lane) was quantified by the adjusted percentage volume—intensity units of pixels of band × mm^2^—after background subtraction using Quantity One Software v4.6.9 (Bio-Rad Laboratories, Hercules CA, USA), and normalized to β-actin (Abcam, Cambridge MA, USA).

### In vitro ubiquitination assay

Human recombinant GST-E1 (50 nM, Boston, Biochem, Cambridge, MA, Cat. #E-306), human recombinant UbcH5c/UBE2D3 (2.5 μM, Boston Biochem, Inc., Cambridge, MA, USA, Cat. #E2-627), human recombinant ubiquitin (250 μM, Boston Biochem, Inc., Cat. #U-100H), human MuRF2 recombinant protein (1 mg, LifeSensors, Cat. #UB305, Malvern, PA, USA), human PPAR-α, -β, and -γ recombinant protein (500 ng, Sigma-Aldrich, Inc., St. Louis, MO, USA, Cat. #SRP2043, Cat. #SRP2044, and Cat. #SRP2045, respectively) were added to reaction buffer (50 mM HEPES, pH 7.5) containing 5 mM MgATP solution (Boston Biochem, Inc., Cat. #B-20) and 0.6 mM DTT then incubated at 37°C for 1 h. The reaction was stopped by adding SDS-PAGE sample buffer and heating, then resolved on a 4–12% Bis–Tris gel with MOPS running buffer (Invitrogen Corp.) and transferred to PVDF membranes for immunoblotting with goat polyclonal anti-MuRF2 antibody (Abcam, Cat. #Ab4387), rabbit polyclonal anti-PPARα antibody (Abcam, Cat. #Ab24509), rabbit polyclonal anti-PPARβ antibody (Millipore, Cat. #AB10094), or rabbit polyclonal anti-PPARγ antibody (Cell Signaling Technology, Cat. #2443).

### Non-targeted metabolomics determination by GC–MS Instrumentation

Cardiac tissue was flash frozen with liquid nitrogen cooled in a biopress, a fraction weighed (~25–30 mg weight), finely ground, and added to fresh 50% acetyl-nitrile, 50% water, and 0.3% formic acid at a standard concentration of 25 mg/475 mcl buffer, then fully homogenized on ice for 10–25 s and placed on dry ice/stored at −80°C. Samples were “crash” deprotonized by methanol precipitation and spiked with D27-deuterated myristic acid (D27-C14:0) as an internal standard for retention-time locking and dried. The trimethylsilyl (TMS)-D27-C14:0 standard retention time (RT) was set at *16.727 min. Reactive carbonyls were stabilized at 50°C with methoxyamine hydrochloride in dry pyridine. Metabolites were made volatile with TMS groups using N-methyl-N-(trimethylsilyl) trifluoroacetamide or MSTFA with catalytic trimethylchlorosilane at 50°C. GC/MS methods generally follow those of Roessner et al. [33], Fiehn et al. [34], and Kind et al. [35], which used a 6,890 N GC connected to a 5,975 Inert single quadrupole MS (Agilent Technologies, Santa Clara, CA, USA). The two wall-coated, open-tubular (WCOT) GC columns connected in series are both from J&W/Agilent (part 122–5512), DB5-MS, 15 m in length, 0.25 mm in diameter, with an 0.25-lm luminal film. Positive ions generated with conventional electron-ionization (EI) at 70 eV are scanned broadly from 600 to 50 m/z in the detector throughout the 45 min cycle time. Data were acquired and analyzed as previously described [36, 37].

### Statistical analysis

Sigma Plot 11.0 and Prism 6.0f  were used to plot and statistically analyze data. Depending upon the experimental design, several statistical tests were applied to the studies. Student’s t test or One Way ANOVA followed by Holm-Sidak pairwise post hoc analysis was performed, indicated in the figure legends. Significance was determined as p < 0.05. Values are expressed as mean ± SE. Statistical analysis on metabolomics data was performed as previously described [36, 37]. Metaboanalyst (v2.0) run on the statistical package R (v2.14.0) used metabolite peaks areas (as representative of concentration) [38, 39]. These data were first analyzed by an unsupervised principal component analysis (PCA), which identified the presence of the MuRF2−/− after 26 weeks high fat diet as the principal source of variance. To sharpen the separation between our three groups, data were next analyzed using a partial least squares discriminant analysis (PLS-DA) to further determine which metabolites were responsible for separating these two groups. The specific metabolites contributing most significantly to the differences identified by PLS-DA between MuRF2−/− and wildtype control group hearts were determined using the variable importance in projection (VIP) analysis in the Metaboanalyst 2.0 environment. The metabolites that best differentiated the groups were then individually tested using the Student’s t-test (Microsoft Excel 2011, Seattle, WA, USA). The VIP and t test significant metabolites were matched to metabolomics pathways using the Pathway Analysis feature in Metaboanalyst 2.0. Heat maps of the metabolite data (individual and grouped) were generated using the GENE E software (http://www.broadinstitute.org/cancer/software/GENE-E/index.html).

## Results

**Fig. 1:**
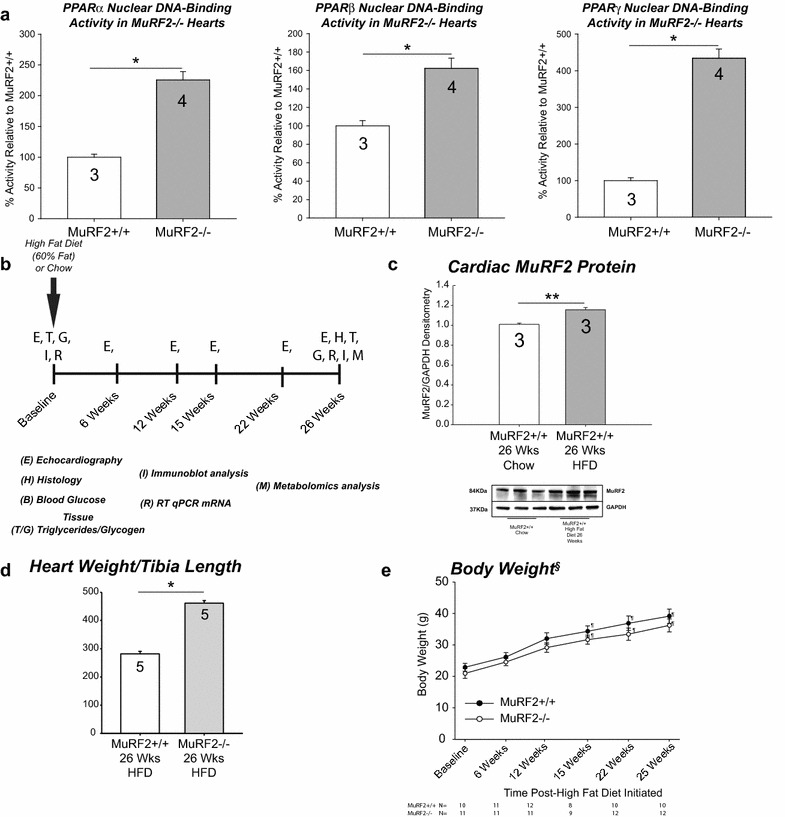
Role of MuRF2 in regulating PPAR isoform activity and its role in high fat diet cardiac hypertrophy in vivo. Isolation of cardiac nuclei from MuRF2−/− and sibling wild type mouse hearts revealed increases in **a** PPAR∝, PPARβ/δ, and PPARγ DNA binding activity using PPRE-DNA as bait and ELISA detection of PPARα protein (N = 4/group). **b** Experimental design of high fat diet (60%)-induced cardiomyopathy. **c** High fat diet induces cardiac MuRF2 levels after 26 weeks HFD (N = 3/group). **d** Endogenous MuRF2 inhibits HFD-induced LV Mass and heart wet weights, as MuRF2−/− hearts have a significant increase in heart weight normalized to body weight and tibia length (N = 5/group). **e** Endogenous MuRF2, found in skeletal muscle and the heart does not affect overall body weight (N indicated below graph). Values expressed as Mean ± SE. Statistical analysis was performed using a Student’s t-test comparing MuRF2−/− and MuRF2+/+ groups. *p ≤ 0.001, **p < 0.01.^ §^p < 0.05 by One Way ANOVA,^ ¶^p < 0.05 vs. MuRF2+/+ baseline by multiple comparisons. $p < 0.05 vs. time-matched MuRF2+/+ (Student’s t-test).

We have recently identified that MuRF2, a muscle-specific ubiquitin ligase, is a critical factor that regulates cardiomyocyte size during development in concert with MuRF1 [40]. MuRF2 has also been described as the effector protein in the Titin-nbr1-p62 complex that responds to mechanical changes in the sarcomere to inhibit transactivation of the nuclear transcription factor serum response factor (SRF) [41]. MuRF2’s regulation of the nuclear specific SRF was the first indication that MuRF2, found primarily in the cytoplasm, could regulate the activity of nuclear receptors, presumably through direct interaction, ubiquitination, and apparent nuclear export [41]. These findings led us to hypothesize that MuRF2 similarly regulates other nuclear receptors critical to cardiomyocytes. To test this, we used MuRF2−/− mice previously characterized without a cardiac or skeletal muscle phenotype [26]. However, we recently identified that MuRF2−/− hearts exhibited changes in metabolomics signatures, indicating that changes in metabolism are present despite any functional effect at baseline [37]. We initially assayed isolated nuclei from MuRF2−/− hearts for their DNA-binding activity contributed by PPARα, PPARβ, and PPARγ as the PPARs have been best described in altering cardiac metabolism [42] and have been reported to be regulated by ubiquitination [43]. To our surprise, we found that MuRF2−/− hearts had significantly increased PPAR activities, with increases in PPARα (+twofold), PPARβ (~1.6 fold), and PPARγ (over +fourfold) activities compared with sibling MuRF2+/+ control mice (Fig. 1a). These findings suggested that endogenous MuRF2 attenuated the activity of all three PPAR transcription factors found in cardiomyocytes. Since MuRF2 is an ubiquitin ligase and PPAR transcription factors have been described with post-translational modification by ubiquitin, we hypothesized that MuRF2 may regulate these PPAR transcription factors in a ubiquitination-dependent manner.

The pathogenesis of diabetic cardiomyopathy involves the enhanced activation of PPAR transcription factors [44]. Diabetic cardiomyopathy is characterized by increased free fatty acid oxidation in parallel with cardiomyocyte insulin resistance [6]. Both the resulting increased fatty acid oxidation and insulin contribute to the decreased ability for the heart to switch away from fatty acid utilization to glucose [45]. Since cardiac MuRF2−/− mice exhibited enhanced PPAR activity, we hypothesized that the induction of diabetic cardiomyopathy would result in both enhanced PPAR activity, resulting in significant cardiac dysfunction compared with wildtype mice. To test this hypothesis, we challenged the MuRF2−/− mice to a 60% high-fat diet, which reproducibly induces insulin resistance and diabetic cardiomyopathy (Fig. 1b) [24].

After 26 weeks of high fat diet challenge, MuRF2−/− mice had significantly lower blood glucose compared with sibling wildtype controls but no differences in serum insulin levels (Additional file 1: Figure S1a). Serum triglycerides were similarly elevated in MuRF2−/− and wildtype controls after 26 weeks of high fat diet (Additional file 1: Figure S1b). Increased cardiac MuRF2 protein levels were identified after high fat diet (Fig. 1c), paralleling MuRF2 increases identified in human inflammatory dilated cardiomyopathy (http://www.ncbi.nlm.nih.gov/geoprofiles/26614376) and coronary artery atherosclerosis (http://www.ncbi.nlm.nih.gov/geoprofiles/16462729). MuRF2−/− hearts increased total weight more than wildtype controls in high fat diet challenge (Fig. 1d) in addition to having significant increases in overall body weight at any 15, 22, and 25 weeks of HFD (Fig. 1e). However, no significant changes were identified in gastrocnemius, soleus, or tibialis anterior muscles weights (Additional file 1: Figure S1c).

**Fig. 2:**
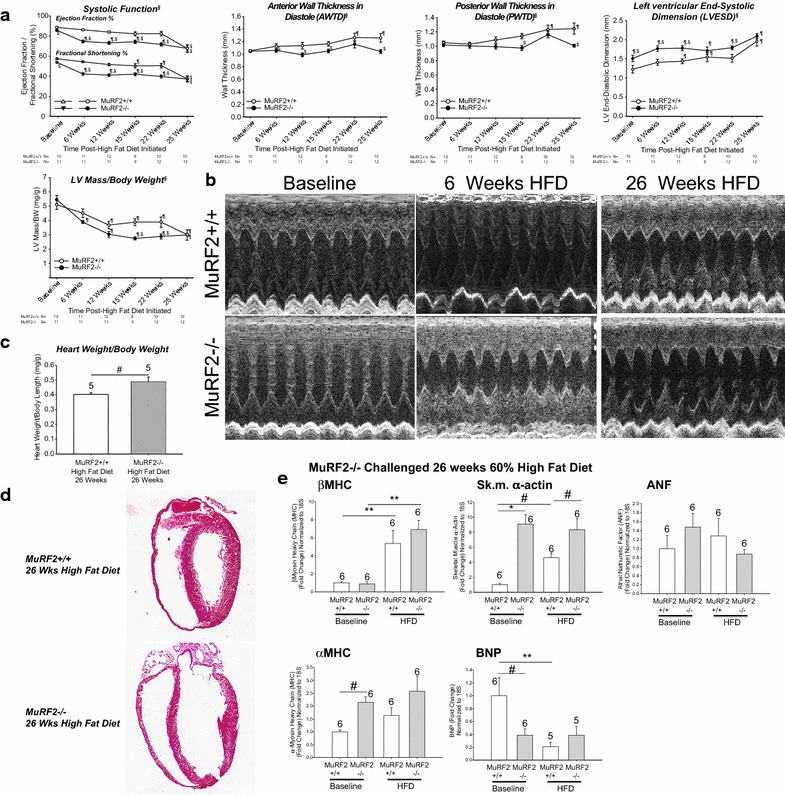
Analysis of MuRF2−/− hearts by conscious echocardiograpy, morphometrics, and heart failure-associated gene expression. **a** MuRF2−/− exhibit an accelerated heart failure by 6 weeks after the initiation of the high fat diet. **b** Representative 2D echocardiographic images from MuRF2−/− hearts at baseline and high fat diet challenge. **c** Endogenous MuRF2 inhibits HFD-induced LV Mass and heart wet weights, as MuRF2−/− hearts have a significant increase in heart weight normalized to body weight and tibia length (N = 5/group). **d** Representative gross histological analysis of MuRF2−/− hearts, found to have wall thinning and increased LV diameters by echocardiological analysis (N = 11 MuRF2+/+, N = 12 MuRF2−/−, see Table 1 and *panel* **a** above). **e** RT-qPCR analysis of heart failure associated fetal gene expression in MuRF2−/− mice at baseline and after 26 weeks high fat diet challenge. Values expressed as Mean ± SE. A One Way ANOVA was performed on echocardiographic studies between all groups, followed by Holm-Sidak (Multiple Comparisons vs. MuRF2 +/+ baseline only). A Student’s t test was then run to compare MuRF2−/− to wildtype control at the same time point.^ §^p < 0.05 by One Way ANOVA,^ ¶^p < 0.05 vs. MuRF2+/+ baseline by multiple comparisons.^ $^p < 0.05 vs. time-matched MuRF2+/+ (Student’s t test). Statistical analysis of heart weight/body weight was performed using a Student’s t test. RT-qPCR analysis analyzed by a One Way ANOVA followed by Holm-Sidak Multiple Comparisons (all pairwise comparisons) *p < 0.001, **p < 0.01,^ #^p < 0.05.

Echocardiographic analysis of MuRF2−/− hearts at baseline found no deficits in function or differences in measurements (Fig. 2a; Table 1) as previously described [40, 46]. Significant deficits in heart function were identified in the MuRF2−/− hearts in as little as 6 weeks after the initiation of high fat diet (Fig. 2a, upper left panel). MuRF2−/− hearts were significantly thinner than MuRF2+/+ hearts from 15–26 weeks of high fat diet feeding (Fig. 2a, upper middle panels, Fig. 2b, d). Both MuRF2−/− and wildtype mice experienced an equal progressive dilation over time on a high fat diet, evidenced by increases in LVESD (Fig. 2a, far right panel). MuRF2−/− hearts exhibited significant dysfunction as early as 6 weeks of HFD compared to MuRF2+/+ hearts (Fig. 2b). MuRF2−/− hearts were significantly larger than MuRF2+/+ hearts after 26 weeks high fat diet (Fig. 2b–d). Diabetic cardiomyocyte-related changes in myosin heavy chain gene expression were next investigated to determine differences between groups. Comparable increases in βMHC were seen in MuRF2−/− and wildtype hearts (Fig. 2e), consistent with previous studies identifying these increases [47]. MuRF2−/− cardiac expression of skeletal muscle α-actin and αMHC were increased in chow control hearts compared to wild type mice, and MuRF2−/− skeletal muscle α-actin was significantly increased as compared to wild type mice after 26 weeks high fat diet (Fig. 2e). No difference existed in αMHC after 26 weeks of high fat diet in either MuRF2−/− or MuRF2 +/+ mice (Fig. 2e), although this is reported in other models of diabetic cardiomyopathy [48, 49]. Brain natriuretic protein (BNP) mRNA was decreased in both MuRF2−/− and controls after 26 weeks high fat diet feeding (Fig. 2e). Taken together, these studies identified that MuRF2−/− hearts failed sooner than MuRF2+/+ hearts, resulting in larger hearts, including LV wall thickness and heart weights after 26 weeks high fat diet challenge.

**Table 1:** Echocardiographic analysis of MuRF2−/− heart function before and after high fat diet challenge

	MuRF2+/+ Baseline, N = 10 (1)	MuRF2−/− Baseline, N = 11 (2)	MuRF2+/+ 6 weeks High Fat Diet N = 11 (3)	MuRF2−/− 6 weeks High Fat Diet N = 11 (4)	MuRF2+/+ 12 weeks High Fat Diet N = 11 (5)	MuRF2−/− 12 weeks High Fat Diet N = 12 (6)	MuRF2+/+ 15 weeks High Fat Diet N = 11 (7)	MuRF2−/− 15 weeks High Fat Diet N = 12 (8)	MuRF2+/+ 22 weeks High Fat Diet N = 10 (9)	MuRF2−/− 22 weeks High Fat Diet N = 12 (10)	MuRF2 +/+ 26 weeks High Fat Diet N = 10 (11)	MuRF2 −/− 26 weeks High Fat Diet N = 12 (12)
AWTS (mm)^§^	1.79 ± 0.04	1.77 ± 0.03	1.82 ± 0.07	1.69 ± 0.05	1.88 ± 0.08	1.63 ± 0.06^$^	1.89 ± 0.05	1.68 ± 0.06^$^	1.87 ± 0.07	1.72 ± 0.07	1.85 ± 0.09	1.61 ± 0.04^$^
LVEDD (mm)	2.85 ± 0.17	3.27 ± 0.16	3.07 ± 0.12	3.05 ± 0.08	2.97 ± 0.09	3.02 ± 0.09	3.08 ± 0.14	2.94 ± 0.12	2.99 ± 0.10	3.05 ± 0.12	3.12 ± 0.10	3.34 ± 0.05
PWTS (mm)^§^	1.65 ± 0.04	1.59 ± 0.03	1.66 ± 0.05	1.42 ± 0.04^$^	1.71 ± 0.08	1.42 ± 0.02$	1.72 ± 0.09	1.65 ± 0.04	1.65 ± 0.04	1.65 ± 0.06	1.65 ± 0.12	1.65 ± 0.05
LV Mass (mg)^§^	105.1 ± 8.1	123.7 ± 7.6	121.4 ± 9.4	111.4 ± 44.2	120.5 ± 7.2	103.9 ± 5.5	153.6 ± 14.5^¶^	102.7 ± 4.9^$^	153.6 ± 15.4^¶^	130.2 ± 7.7^¶^	156.6 ± 12.5^¶^	125.7 ± 4.1^$^
LV Vol;d (μl)	32.5 ± 4.7	44.6 ± 4.8	37.9 ± 3.8	37.0 ± 2.4	34.6 ± 2.6	36.2 ± 2.5	38.7 ± 4.0	34.40 ± 3.6	37.1 ± 3.7	35.4 ± 2.9	39.1 ± 3.2	45.5 ± 1.7
LV Vol;s (μl)^§^	4.0 ± 0.9	6.5 ± 0.8	5.5 ± 0.8	9.6 ± 0.9^¶,$^	5.6 ± 0.7	9.7 ± 0.8^¶,$^	7.3 ± 1.4	9.1 ± 1.4^¶^	6.7 ± 1.1	9.8 ± 0.7^¶,$^	12.9 ± 1.9^¶^	14.6 ± 1.1^¶^
BW (g)^§^	20.9 ± 1.6	22.9 ± 1.2	24.5 ± 1.2	26.2 ± 1.4	29.1 ± 1.5^¶^	32.0 ± 1.8^¶^	31.7 ± 1.4^¶^	34.3 ± 1.7^¶^	33.4 ± 1.9^¶^	36.9 ± 2.3^¶^	36.2 ± 2.1^¶^	39.1 ± 2.3^¶^
HR (bpm)^§^	609 ± 19	590 ± 18	671 ± 8^¶^	636 ± 8^$^	658 ± 13^¶^	654 ± 14^¶^	681 ± 1^¶^	666 ± 13^¶^	665 ± 11^¶^	648 ± 13	667 ± 12^¶^	668 ± 9^¶^

High-resolution transthoracic echocardiography performed on conscious MuRF2−/− and age-matched wild type mice at baseline, 6, 12, 15, 22, and 26 weeks high fat diet. Data represent mean ± SEM. A One Way ANOVA was performed between all groups, followed by Holm-Sidak Multiple Comparisons vs. MuRF2+/+ baseline. A Student’s t test was then run to compare MuRF2−/− to wildtype control at the same time point.

*HR* heart rate, *ExLVD* external left ventricular diameter, *bpm* heart beats per minute, *AWTD* anterior wall thickness in diastole, *AWTS* anterior wall thickness in systole, *PWTD* posterior wall thickness in diastole, *PWTS* posterior wall thickness in systole, *LVEDD* left ventricular end-diastolic dimension, *LVESD* left ventricular end-systolic dimension, *FS* fractional shortening, calculated as (LVEDD-LVESD)/LVEDD × 100, *EF*% ejection fraction calculated as (end Simpson’s diastolic volume − end Simpson’s systolic volume)/end Simpson’s diastolic volume × 100, *ND* not determined.

^§^ p < 0.05 by One Way ANOVA.

^¶^ p < 0.05 vs. MuRF2+/+ Baseline by Multiple Comparisons.

^$^ p < 0.05 vs. time-matched MuRF2+/+ (Student’s t test).

**Fig. 3:**
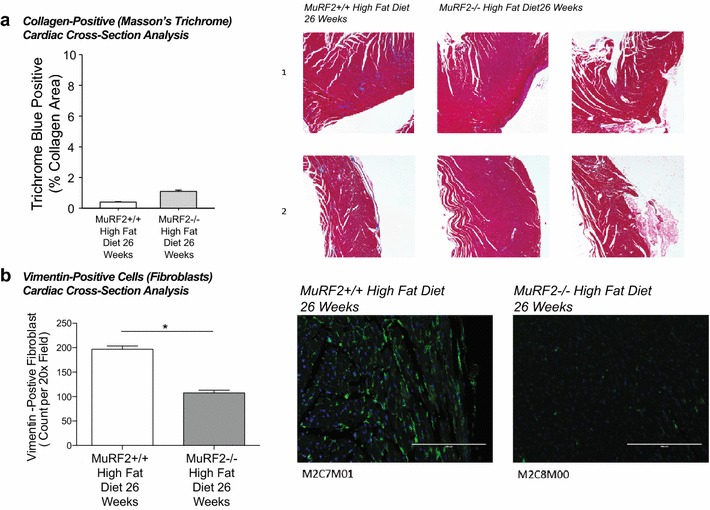
Histological analysis of cardiac fibrosis. **a** Fibrosis analysis of Masson’s Trichrome-stained heart sections of MuRF2−/− and wild type hearts after 26 weeks high fat diet reveals no significant differences. **b** Confocal immunofluorescence analysis of vimentin (fibroblasts) in cardiac cross-sections from MuRF2−/− mice after 26 weeks HFD (N = 3/group). Values expressed as Mean ± SE. Statistical analysis was performed using a Student’s t test. *p < 0.001, **p < 0.01, ^#^p < 0.05.

LV remodeling is a distinctive finding in the pathogenesis of diabetic cardiomyopathy. These changes include the development of fibrosis, resulting from the accumulation of extracellular collagen [50, 51]. Reduced MMP2 activity [52] and O-GlcNAcylation stimulated cardiac fibroblast collagen synthesis has been reported [53]. In this particular model, less than 2% fibrosis was identified throughout the heart in MuRF2−/− and wildtype controls (Fig. 3a). However, MuRF2−/− hearts revealed a parallel reduction in vimentin-positive fibroblasts (Fig. 3b). Throughout the course of the study, only one mouse died at 21 weeks of high fat diet. This wildtype mouse, interestingly, revealed almost 4% fibrosis (Additional file 2: Figure S2c) with amorphous waxy infiltrates and leukocyte infiltrates (Additional file 2: Figure S2b) not seen in either MuRF2−/− or wildtype hearts after 26 weeks high fat diet (Additional file 2: Figure S2a). Overall, while MuRF2−/− hearts have significant increases in fibrosis, the total fibrosis is minimal and does not account for the large changes in cardiac size, dysfunction, and suggests other non-structural signaling pathways likely are involved in the MuRF2−/− exaggerated cardiac dysfunction in diabetic cardiomyopathy.

**Fig. 4:**
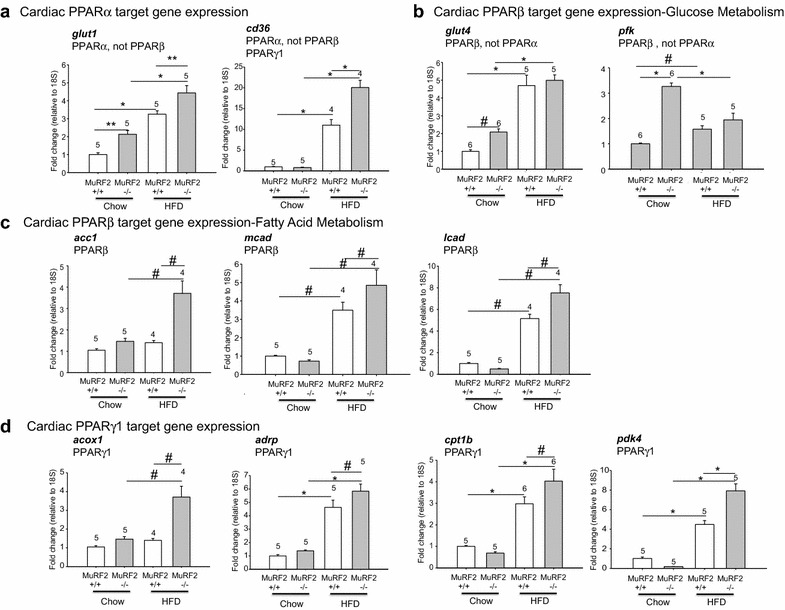
High fat diet-induced increases in PPAR-regulated gene (mRNA) levels are exaggerated in cardiac MuRF2−/− hearts. RT-qPCR analysis of cardiac mRNA of genes identified as PPAR isoform “specific” based on cardiac transgenic PPARα, PPARβ, and PPARγ1 studies as described in the text. **a** Cardiac PPARα target gene expression, **b** PPARβ-regulated mRNA target genes involved in glucose metabolism, **c** PPARβ-regulated mRNA target genes involved in fatty acid metabolism. **d** PPARγ1-regulated mRNA target genes. Values expressed as Mean ± SE. Values expressed as Mean ± SE. The significance of observed differences in grouped mean values was determined using a One Way ANOVA followed by Holm-Sidak pairwise post hoc analysis. N per group indicated above graph. *p ≤ 0.001, **p < 0.01,^ #^p < 0.05.

Cardiac PPARα, PPARβ, and PPARγ1 have pivotal roles in the pathophysiology of diabetic cardiomyopathy [44]. Therefore, we next investigated the expression of cardiac PPAR isoform regulated genes previously described in vivo [54–56]. Gene expression of the cardiac PPARα target genes (not regulated by cardiac PPARβ i.e. *glut1* and *cd36*) (Fig. 4a), cardiac PPARβ target genes associated with glucose metabolism (not regulated by cardiac PPARα, i.e. *glut4*, *pfk*, *acc1*, *mcad,* and *lcad*) (Fig. 4b, c), and cardiac PPARγ1-regulated cardiac genes (i.e. *acox1*, *adrp*, *cpt1b*, *and pdk4*) (Fig. 4d) were evaluated in MuRF2−/− mouse hearts. Notably, MuRF2−/− hearts challenged with high fat diet exhibited significantly increased levels of PPARα-regulated genes (Fig. 4a), PPARβ-regulated genes associated with fatty acid metabolism (Fig. 4c), and PPARγ1-regulated genes (Fig. 4d). MuRF2−/− hearts did not differ from MuRF2+/+ hearts in PPARβ-regulated target genes associated with glucose metabolism (*glut4* and *pfk*, Fig. 4c). Like the PPAR isoform activities assays of the MuRF2−/− heart nuclei demonstrated, MuRF2−/− hearts exhibited enhanced PPAR activities. At the mRNA level, MuRF2−/− hearts exhibited significant increases in PPARα compared with wildtype mice, but no differences in PPARβ or PPARγ1 (Additional file 3: Figure S3).

**Fig. 5:**
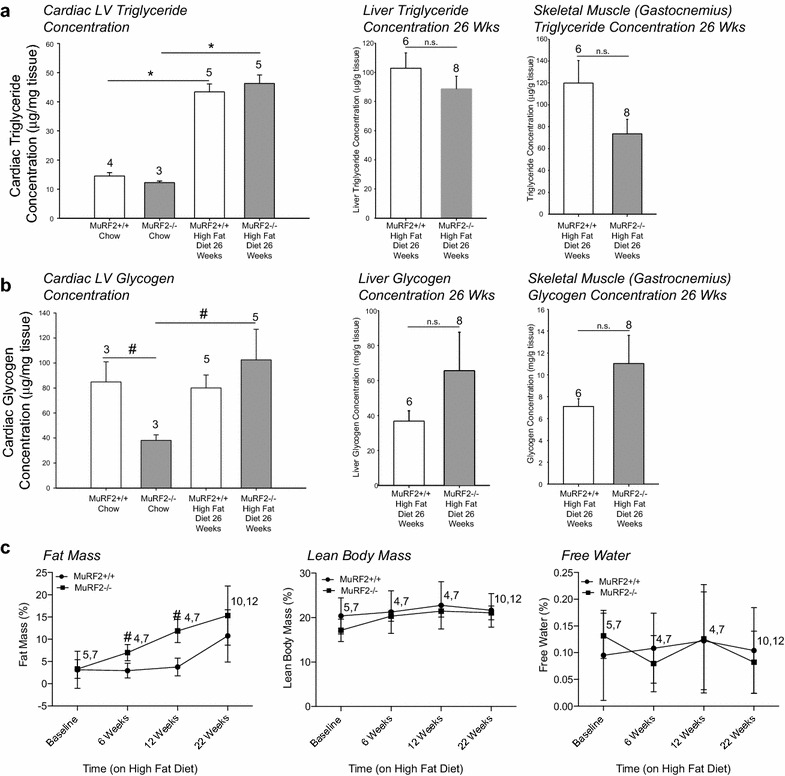
Analysis of tissue triglyceride, glycogen, and fat mass in MuRF2−/− mice after high fat diet challenge. **a** Triglyceride analysis of cardiac left ventricle (LV), liver, and skeletal muscle (gastrocnemius). **b** Glycogen analysis of cardiac LV, liver, and skeletal muscle (gastrocnemius). **c** Magnetic resonance imaging (MRI) analysis of fat mass, lean body mass, and free water at baseline, 6, 12, and 22 weeks HFD. Values expressed as Mean ± SE. A one-way ANOVA was performed to determine significance of cardiac LV triglyceride and glycogen concentrations, followed by a Holm-Sidak pairwise comparison to determine significance between groups. A Student’s t test was performed comparing MuRF2−/− vs. MuRF2+/+ groups in all other studies. Numbers above bars represent number of animals (N) included in each experiment (N = MuRF2+/+, MuRF2−/− in **c**). *p < 0.001, **p < 0.01,^ #^p < 0.05.

Fatty acids are the primary fuel of the heart in addition to being ligands for the PPAR transcription factors. High fat diets have been reported to increase cardiac triglyceride content [57]. The increased storage fat (as myocardial triglyceride) that occurs in the development of type 2 diabetic cardiomyopathy has been hypothesized as one mechanism that free fatty acids are toxic to the heart [58–60]. The mishandling of cardiac glycogen is also a frequent manifestation of diabetic cardiomyopathy [61]. We hypothesized that increased levels of fatty acid in the MuRF2−/− hearts could contribute to the enhanced heart failure they demonstrated in diabetic cardiomyopathy. Since MuRF2 has been reported in the heart and skeletal muscle, in addition to the liver, we measured cardiac triglycerides and glycogen after 26 weeks of high fat diet to determine if alterations in these storage forms of fat and glucose could be contributing to the increased heart weight or dysfunction in the MuRF2−/− hearts (Fig. 5). Compared to the control feeding, both MuRF2−/− and MuRF2+/+ hearts had increased cardiac triglyceride levels (Fig. 5a). However, MuRF2−/− hearts did not have significantly different triglyceride levels compared with wildtype after 26 weeks high fat diet feeding. MuRF2−/− liver and skeletal muscle after 26 weeks high fat diet feeding was similarly not significantly different from wildtype controls (Fig. 5a). MuRF2−/− hearts from dietary controls (chow) had significantly decreased cardiac glycogen compared with wildtype hearts (Fig. 5b). While MuRF2−/− hearts accumulated significantly increased glycogen after 26 weeks high fat diet, the increases MuRF2−/− liver and skeletal muscle accumulated did not reach significance (Fig. 5b). Together, these studies illustrate that the MuRF2−/− hearts are able to store fat (as triglyceride), but have alterations in glycogen storage capacity both at steady state (baseline) conditions and after high fat diet challenges. Akt and glycogen synthase kinase (GSK)-3β are reported to be decreased in diabetic cardiomyopathy, along with increases in fibrosis and inflammation [48, 62].

Recent studies have demonstrated a role for PPAR activation in developing adiposity and weight gain in models of diabetes. In one study, treatment with rosiglitazone in mouse models of diabetes was shown to promote increases in cardiac size and enhanced fat volume [63]. Similarly, rosiglitazone side effects in patients have revealed increasing fat gain [64]. At baseline, MuRF2−/− mice had comparable fat and lean body mass as wildtype controls (Fig. 5c). However, during the development of insulin resistance, MuRF2−/− mice demonstrated significantly more fat mass at 6 and 12 weeks of high fat diet, but no changes in lean body mass (Fig. 5c). While the specific mechanisms by which rosiglitazone regulates fat mass is not completely clear, the enhanced PPAR activities seen in MuRF2−/− mice may be one contributing factor to the accumulation of fat mass during which cardiac function is significantly worse than wildtype mice challenged in parallel with a high fat diet.

The post-translational modification of intracellular proteins by O-linked N-acetylglucosamine (O-GlcNAc) in diabetes is a result of the excess glucose that drives the reaction. O-GlcNAc, in concert with ubiquitin, mediates several aspects of diabetic cardiomyopathy [53, 65–68]. O-GlcNAc modified proteins impair cardiomyocyte calcium cycling via its direct effects on phospholamban [68, 69]. O-GlcNAcylation also blunts autophagy, down regulates Nkx2.5 expression, and stimulates cardiac fibroblast collagen synthesis to mediate cardiac dysfunction [53, 65, 66]. Therefore, we measured the amount of O-GlcNAc proteins in MuRF2−/− heart, hypothesizing that the loss of MuRF2 cleared fewer O-GlcNAc-modified proteins, to mediate the enhanced cardiomyopathy seen in vivo. Immunoblot analysis of O-GlcNAc-modified proteins in MuRF2−/− hearts demonstrated no differences from wildtype hearts when mice were fed a chow diet or 26 weeks of high fat diet (Additional file 4: Figure S4). While modest increases in O-GlcNAc levels were identified after 26 weeks of high fat diet, as expected with the observed hyperglycemia, differences in O-GlcNAc could did not appear to contribute to the exaggerated MuRF2−/− cardiac dysfunction.

Since NF-κB signaling, defective insulin signaling, JNK signaling, and alterations in autophagy have been implicated in the pathogenesis of diabetic cardiomyopathy [19, 70–72], we next determined if MuRF2−/− hearts had alterations in these processes that may explain their more severe phenotype. After 26 weeks of high fat diet, MuRF2−/− hearts did not exhibit enhanced NF-κB activity (determined by p-p65 western blot), decreased IRS-1 signaling (determined by p-IRS-1 western blot), or alternations in JNK signaling (determined by p-cJun) (Additional file 5: Figure S5a). Similarly, measures of cardiac autophagy in MuRF2−/− hearts after high fat diet did not differ from wildtype controls, including autophagy flux (LC3II/LC3I proteins ratio post-bafilomycin treatment by western blot), p62, or VPS34 protein levels by western blot (Additional file 5: Figure S5b). These studies demonstrated that the more severe MuRF2−/− phenotype was not due to alterations in NF-κB, insulin, or JNK signaling or reductions in autophagy that have been reported to result in more severe diabetic cardiomyopathy [19].

Evidence from a variety of cell culture studies have implicated ubiquitin as a post-translational modifier of PPAR transcription factors and their coreceptors/co-activators [43]. These have been in liver, lung, fibroblast, adipocytes, and macrophage (as recently reviewed [43]). These studies have found that the ubiquitin-mediated inhibition of PPAR isoforms PPARα, PPARβ, and PPARγ are: 1) ligand-dependent (ligand is required for ubiquitination and/or degradation to occur); and 2) the ratio of ubiquitin ligase (e.g. MDM2 [73]) determines activation (e.g. MDM2:PPARα ratio < 1) or inhibition (e.g. MDM2:PPARα > 1 [73]). Since considerable evidence shows that MuRF2−/− hearts enhance PPAR-activity suggesting that endogenous cardiac MuRF2 inhibits PPAR activities by nuclear PPRE-binding (Fig. 1a) and PPAR-regulated gene expression (Fig. 4), we next focused on how the muscle-specific ubiquitin ligase MuRF2 might exert its inhibitory effects based on our current knowledge of how ubiquitin regulates PPAR in cancer cells.

**Fig. 6:**
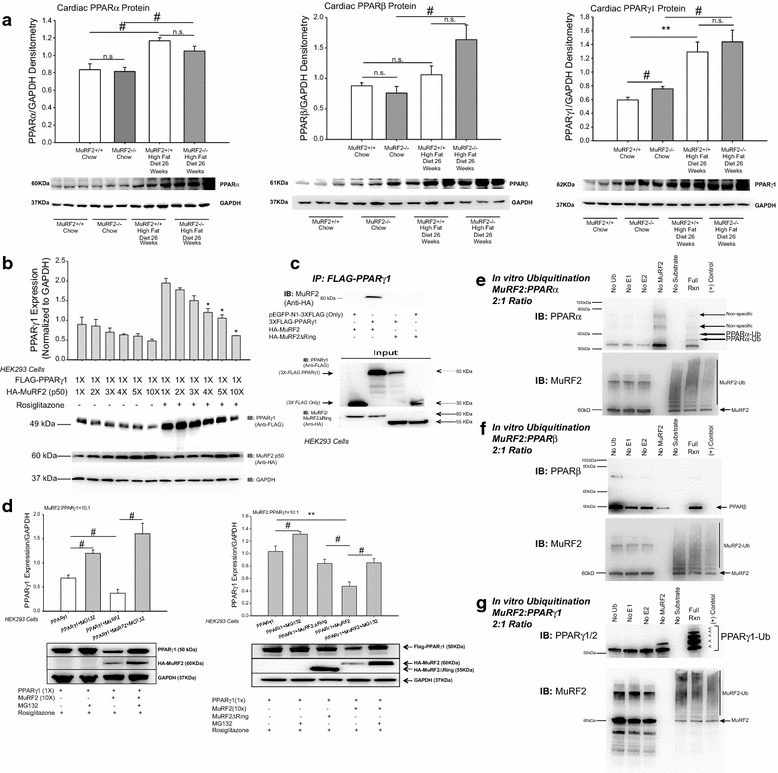
The ratio of MuRF2 to PPARγ1 determines the ubiquitin post-translational modification and ligand-dependent protein levels. **a** Immunoblot analysis of cardiac LV PPARα, PPARβ, and PPARγ1 levels normalized to GAPDH. N = 3/group. **b** Increasing MuRF2 results in a PPARγ1 ligand (Rosiglitazone)-dependent decrease in PPARγ1 in vitro 24 h after transfection. HEK293 cells were co-transfected with MuRF2 and PPARγ1 (as indicated below graph). After 24 h, 1 microM Rosiglitazone was added overnight and cells harvest at 48 h. *p < 0.05 vs. MuRF2:PPARγ1 ratio of 1:1 without Rosiglitazone. **c** Immunoprecipitation studies identifying MuRF2 interaction with PPARg1. HEK293 cells were transfected p3XFlag-PPARγ1 (or p3XFlag-Empty vector), pcDNA3.1-HA-MuRF2p50a (or HA-MURF2∆Ring) and immunoblotted for MuRF2 (anti-HA). **d** *Left* Proteasome inhibition with MG132 prevents MuRF2’s degradation of PPARγ1 in a *Right* ligase-dependent (Ring Finger-dependent) manner. HEK293 cells transfected with p3XFlag-PPARγ1, pcDNA3.1-HA-MuRF2p50a and treated with MG132 (50 μM) for 2.5 h before Rosiglitazone added (1 μm). **e**–**g** In vitro ubiquitination assays of MuRF2’s ability to ubiquitinate PPARα (**e**), PPARβ (**f**), and PPARγ1 (**g**), with all lanes having Ub, E1, E2, MuRF2, and PPAR (=full reaction), unless otherwise indicated. Immunoblot for MuRF2 illustrates auto-ubiquitination (=MuRF2 activity) present in the same reaction as mono-ubiquitination (PPARα) and poly-ubiquitination (PPARγ1). Values expressed as Mean ± SE of three independent experiments. A one-way ANOVA was performed to determine significance, followed by a Holm-Sidak pairwise comparison to determine significance between groups.^ #^p < 0.05, **p < 0.01.

Like other ubiquitin ligases, MuRF2 interacts with a number of protein substrates. Notably, MuRF2 and MuRF1 redundantly interact with roponin-I (TnI), TnT, myosin light chain 2, and T-cap (telethonin) in yeast two-hybrid studies [74]. Unlike MuRF1, MuRF2 has not been shown to degrade any of these substrates (as recently reviewed [75] ). But critical regulation of microtubule, intermediate filament, and sarcomeric M-line stability during striated muscle development [22] and regulation of E2F activity [40]. Understanding that high fat diet induced MuRF2 expression, we next identified PPARα, PPARβ, and PPARγ1 (as the PPARγ2 isoform is restricted to adipocytes) (Fig. 6a). Interestingly, in steady state conditions, cardiac PPARα and PPARα protein levels in MuRF2−/− mice did not differ compared with wildtype controls. However, PPARγ1 levels were slightly (and significantly) increased at baseline (Fig. 6a, far right). After challenge with PPAR ligands (free fatty acids from high fat diet) for 26 weeks, no differences in MuRF2−/− cardiac PPARα and PPARγ1 were identified by immunoblot analysis, but a significant increase in PPARβ protein expression was identified (Fig. 6a). Taken together, these studies illustrate that the steady state levels of cardiac PPARα and PPARγ1 isoforms are not affected by the presence of MuRF2 or its increase (Fig. 1c) after high fat diet challenge. Moreover, these results suggest that MuRF2’s changes in PPARα and PPARγ1 activities could be due to one of the multiple non-canonical post-translational modifications by ubiquitin (e.g. mono-ubiquitination) that are not associated with proteasome dependent and degradation. How MuRF2 is regulating PPARβ without being able to ubiquitinate it directly (Fig. 6f) is unclear. But the mechanism would be indirect include the possibility that MuRF2 it targeting the inhibition of a yet to be determined ubiquitin ligase(s) that normally degrades PPARβ. For example, PPARβ in cancer cells (HEK293 and NIH3T3) is ubiquitinated and degraded in a ligand (GW501516)-dependent manner [76]. While the identification of the ubiquitin ligase targeting PPARβ is not known at this time, ubiquitin ligases degrading other isoforms (e.g. PPARγ) have been reported in adipocytes (MKRN1) [77]. Conversely, MuRF2 ubiquitination could be enhancing a de-ubiquitinase (DUB) that prevents proteasome-mediated degradation by this unidentified E3(s).

We next sought to determine the underlying mechanism by which endogenous MuRF2 exerted inhibition on PPAR-regulated genes (Fig. 4). Based in the limited work performed on PPAR ubiquitination (as recently reviewed [43]), we hypothesized that the ratio of MuRF2 to the substrate may regulate whether the protein was degraded in a proteasome-dependent manner, as previously reported in cancer with MDM2:PPARα ratios [73]. Increasing the MuRF2:PPARγ1 ratios resulted in a dose-dependent decrease in steady state protein levels, consistent with poly-ubiquitination and subsequent degradation (Fig. 6b). To determine the role of the proteasome in this process, we next repeated these experiments and found that the MuRF2-mediated decrease in PPARγ1 could be prevented by adding the proteasome inhibitor MG132 (Fig. 6d). Since previous studies have reported that ubiquitin ligase mediated proteasome degradation of PPARs is ligand dependent (as recently reviewed [43]), we next repeated these studies in the presence and absence of PPARγ ligand rosiglitazone, demonstrating that MuRF2’s dose-dependent degradation of PPARγ1 was ligand dependent (Fig. 6d). To establish that MuRF2 interacts with PPARγ1, we performed immunoprecipitation studies by co-transfecting cells with HA-MuRF2 or HA-MuRF2∆Ring (lacking the ubiquitin ligase region) and FLAG-PPARγ1 (Fig. 6c). Immunoprecipitating PPARγ1, we identified that MuRF2 bound PPARγ1 by immunoblots (Fig. 6c). Unexpectedly, MuRF2∆Ring did not bind PPARγ1 in parallel studies suggesting MuRF2’s Ring Finger domain has structural importance in the interaction with PPARγ1.

In vivo, the cardiac MuRF2 protein levels increased ~30% in wild type mice (Fig. 1c), while steady state levels of PPARα, PPARβ, and PPARγ1 were either increased (PPARα, PPARγ1) or unchanged (PPARβ) in wildtype mice in response to 26 weeks of high fat diet compared to chow-fed wildtype controls (Fig. 6a). With no evidence that cardiac MuRF2 affected steady state PPARγ1 isoform protein levels yet inhibited PPARγ1 activity in vivo (MuRF2−/− hearts had enhanced PPARγ1 activities), we next tested how MuRF2 may be inhibiting PPARγ1 mechanistically. Specifically, we wanted to determine why the physiological relevance of MuRF2-mediated degradation (with MuRF2:PPARg1 at levels 10:1) in vivo did not appear relevant in the context of diabetic cardiomyopathy. The experimental studies indicating that high MuRF2:PPARγ1 ratios resulted in ligand-dependent proteasome degradation may be relevant in other disease processes where MuRF2 levels are increased more in vivo. However, such a disease process has not been described to date. Therefore, we focused our studies of MuRF2-mediated ubiquitination of PPAR isoforms using ratios of 2:1 (Fig. 6e–g). Whereas, MuRF2 did not appear to add poly-ubiquitination leading to degradation, MuRF2 drove multi-mono-ubiquitination on PPARα and PPARγ1. Specifically, MuRF2 di-mono-ubiquitinated PPARα, while adding ~four ubiquitin moieties to PPARγ1 (Fig. 6e, g, respectively). PPARβ, however, was unexpectedly not modified by ubiquitin at all in vitro. These studies add perspective to our initial findings that endogenous cardiac MuRF2 had the greatest regulation of PPARγ, with MuRF2−/− hearts exhibiting 400%+ PPARγ activity found in the sibling wildtype hearts (Fig. 1a, far right). Endogenous MuRF2 similarly had the next most inhibition of PPARα, with MuRF2−/− hearts exhibiting ~250% PPARα activity found in sibling wildtype hearts (Fig. 1a, far left). Quite surprising was the finding that MuRF2 did not ubiquitinate PPARβ (Fig. 6f), despite MuRF2 hearts exhibiting 80% more activity than wildtype controls (Fig. 1a, middle).

**Fig. 7:**
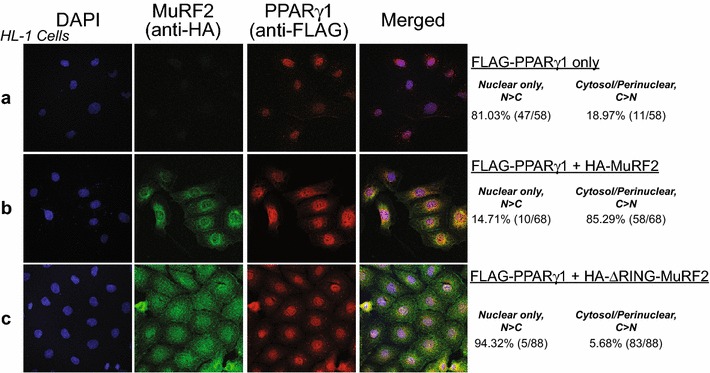
MuRF2 alters nuclear localization of PPARγ1 in a ubiquitin ligase region (RING)-dependent manner without altering steady state protein levels (as found in vivo). Increased MuRF2 alters PPARγ1 localization from primarily nuclear **a** to increased cytosolic/perinuclear localization **b** in HL-1 cardiomyocyte-derived cells. MuRF2 lacking ubiquitin ligase activity (∆RING FingerMuRF2, **c** does not alter PPARγ1 localization compared controls (*top row*), indicating MuRF2’s dependence on its ubiquitin ligase activity in mediating these changes. Representative of three biological replicates. *Right* Percentages based on the number of cells analyzed (N = 58, 68, and 88 in FLAG-PPARγ1, FLAG-PPARγ1 + HA-MuRF2, and FLAG-PPARγ1 + HA-∆RING-MuRF2 groups, respectively).

To gain more insight on how MuRF2 may be inhibiting transcriptional activity by ubiquitination, we next performed nuclear localization studies using confocal microscopy (Fig. 7). In control cells, we found that PPARγ1 could be found in both the nucleus and cytosol, with most cells having primarily nuclear localization (81%) (Fig. 7a). Increasing MuRF2 (2:1 ratio of PPARγ1 transfected) interestingly resulted in an increase in the “perinuclear” localization of PPARγ1 (Fig. 7b). Notably, MuRF2 co-localized to these perinuclear regions (Fig. 7b). Parallel studies using the MuRF2 without its ubiquitin ligase activity (∆RING-MuRF2) abrogated the perinuclear targeting of PPARγ and colocalization with MuRF2 (Fig. 7c). Since we demonstrated that MuRF2, but not MuRF2∆Ring, bound to PPARγ1 (Fig. 6c), these studies indicates that MuRF2 regulation of PPARγ1 location may lie in its ubiquitin ligase activity and/or through some structural role required for interaction since MuRF2’s Ring Finger domain is required to bind PPARγ1 (Fig. 6c). Taken together, these studies suggested that MuRF2 targets an ubiquitin-mediated regulation of PPARγ1 activity by altering its localization within the nucleus, paralleling recent studies demonstrating autophagic sequestration of receptors in the endoplasmic reticulum and nucleus [78].

**Fig. 8:**
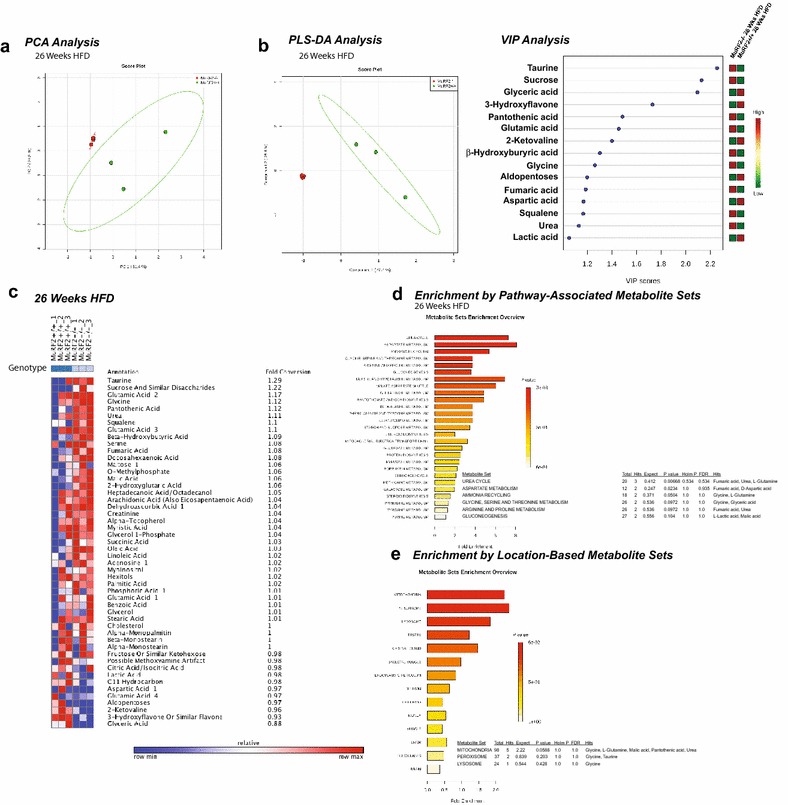
Non-targeted cardiac metabolomics of MuRF2−/− mice after 26 weeks challenge with high fat diet. **a** Principal components analysis, **b** Partial least squares-discriminant analysis (PLS-DA) and variable importance in the projection analysis, **c** Heat map of metabolites identified by non-targeted GC/MS analysis of cardiac tissue. **d** Enrichment by pathway-associated metabolite sets and **e** location-Based Metabolite sets determined from VIP significant and t test significant metabolites identified. N = 3/group. Significance determined as p < 0.05.

By non-targeted metabolomics analysis, MuRF2 hearts had significant decreases in taurine, myoinositol, and four metabolites involved in malate-aspartate shuttle (glycerol-1phosphate, urea, malic acid, and phosphoric acid) [37]. In the present study, we similarly analyzed MuRF2−/− hearts using non-targeted metabolomics analysis after 26 weeks of high fat diet (Fig. 8). The separation of MuRF2−/− hearts from wildtype was clear using Principal Components Analysis (PCA) (Fig. 8a) as well as Partial Least Squares Discriminant Analysis (PLS-DA) and Variable Interdependent Parameters (VIP) analysis (Fig. 8b). Among all of the annotated metabolites (Fig. 8c), the VIP significant analytes detected were taurine, sucrose, glyceric acid, 3-hydroxyflavone, pantothenic acid, and glutamic acid, among others (Fig. 8b). Enrichment analysis identified the (1) urea cycle; (2) aspartate metabolism; and (3) taurine and hypotaurine metabolism to be the highest fold enriched by metabolite sets (Fig. 8d). Based on location, the mitochondria, peroxisome, and lysosome were the most enriched (Fig. 8e). Pathway analysis identified (1) taurine and Hypotaurine metabolism; (2) glycine, serine, and threonine metabolism; and (3) alanine, aspartate, and glutamate metabolism as the pathways most significantly affected when both t test and VIP significant metabolites were analyzed (Additional file 6: Figure S6).

## Discussion

While the present study demonstrates a role for the muscle specific ubiquitin ligase in regulating PPAR transcription factors in the context of diabetes, the clinical implications of these findings are broader. Recent studies have identified reductions in MuRF2 in patients with diabetic ischemic heart failure (GEO ID: 87403976, http://www.ncbi.nlm.nih.gov/geoprofiles?term=87403976). Rare MuRF2 mutations in patients with familial hypertrophic cardiomyopathy have also been identified that are associated with a greater LV wall thickness than those without MuRF2 mutations [79]. Genome wide identification of SARS-CoV susceptibility loci using the collaborative cross has identified MuRF2 as a susceptibility factor to SARS infection, including evidence that MuRF2−/− mice are more susceptible (R.S. Baric, personal communication and GEO accession no. GSE64660, http://www.ncbi.nlm.nih.gov/geo/query/acc.cgi?acc=GSE64660). Taken together, these studies suggest a role for MuRF2 in an increased susceptibility to disease. Given the growing evidence that PPARs have anti-inflammatory activity by competitively inhibiting NF-κB and AP1 (cJun/cFos) experimentally (recently reviewed by Lockyer et al. [80]), it is possible that reduced (or ablated) MuRF2 in patients may increase their susceptibility to infection in addition to diabetes as we demonstrate in the present study.

The dynamic regulation of PPAR transcription factor activity reflects the complexity found routinely in biology. PPAR family transcription factors routinely partner with RXR to regulate transcription of target genes, but co-factors such as PGC-1a enhance PPAR activity (induced by exercise) and mitochondrial biogenesis, which nuclear receptor corepressor 1 (NCOR1) antagonizes these activity (as recently reviewed by Fan and Evans) [81]. PPAR activity is also regulated by ligands which bind the ligand binding domain and include endogenously free fatty acids, eicosinoids, and environmental compounds [82]. In addition to the various co-complex partners that regulate PPARs, there are other transcription factors that compete with PPARs to regulate their activity, notably NF-κB, making enhanced PPAR activity an anti-inflammatory state [80]. It is on top of this complexity that post-translational modification by ubiquitin and SUMO has been reported primarily in cancer cells, and recently by our group in cardiomyocytes for the first time [83]. In our recent review on this topic, few ubiquitin ligases have been described, but ubiquitination and proteasome-dependent degradation of PPAR as well as SUMOylation has been reported in PPARα, PPARβ, and PPARγ1 in primarily non-physiological cultured conditions. The report here of the first ubiquitin ligase regulating PPARγ1 by ubiquitination in a physiological process and cardiac pathophysiological process (diabetes) adds detail to a dimension of regulation we’ve just begun to understand.

These layers of complexity are apparent in the present study. While our initial studies revealed that MuRF2−/− hearts had enhanced PPARα, PPARβ, and PPARγ1 (most prominently PPARγ1) activity measured by binding of PPRE DNA (Fig. 1a). However, these enhanced activities were clearly not due to increased total protein levels of PPARα and PPARγ1 (Fig. 6a). Similarly, PPARα and PPARγ1 were multi-mono-ubiquitinated as a mechanism to explain their inhibition (Fig. 6e, g), while PPARβ was not ubiquitinated by MuRF2 (Fig. 6f). Like previous studies from our laboratory, the nuclear localization after MuRF protein post-translational ubiquitination may be regulating this process with PPARγ1 (Fig. 7) [83]. However, the indirect regulation of PPARβ protein levels (enhanced in MuRF2−/− hearts) is complex. The increase in PPARβ protein levels is not transcriptionally regulated (Additional file 3: Figure S3b), leading to our hypothesis that MuRF2 regulates PPARβ through complex post-translational mechanism(s) as described above (e.g. MuRF2 degradation of E3(s) targeting PPARβ or MuRF2 enhancing DUBs targeting PPARβ). Similarly complexity may lie with MuRF2’s regulation of PPARα as PPARα mRNA levels are enhanced in MuRF2−/− hearts throughout this study (Fig. 3a). The regulatory elements in the promoter region of PPAR include activator protein-1 (AP-1) and how MuRF2 may inhibit these proteins is not clear [84, 85]. MuRF2−/− mice have equivalent PPARγ1 protein (Fig. 6a) and PPARγ1 mRNA (Additional file 3: Figure S3c). However, MuRF2−/− hearts also have the most highly activated PPARγ1 activity by several measures (Figs. 1a, 4d), indicating that the strong post-translational multi-mono-ubiquitination in vitro (Fig. 6g) may be MuRF2’s primary mechanism of regulating PPARγ1 in vivo.

MuRF2 has previously been shown to regulate two nuclear transcription factors found in myocytes, paralleling its role in regulating nuclear PPAR isoforms in the present study. Initial studies investigating the role of MuRF2 in differentiated myocytes identified that MuRF2 binds critical signaling regions of the giant protein titin (via titin kinase region), to interact and regulate the activity and localization of the nuclear transcription factor SRF [41]. However, cardiac MuRF2 did not appear to regulate SRF activity in vivo, when stimulated by known SRF activating pressure overload-induced cardiac hypertrophy [26], suggesting this regulation is disease context specific. Subsequently, MuRF2 has been implicated in redundantly regulating (with MuRF1) other nuclear transcription factors implicated in developmental physiological hypertrophy [40]. In both cases, no evidence of MuRF2-mediated degradation was identified, consistent with the degradation-independent regulation of PPAR isoforms in the present study. The most commonly reported regulation of PPAR isoforms from the cancer literature has identified that post-translational modification with ubiquitin and SUMO appears to regulate PPAR most commonly to inhibit their regulation.

The post-translational regulation of PPAR by ubiquitin has previously been identified in cancer cells. However, ubiquitin-regulation of PPARs in the heart has not. Similarly, specific ubiquitin ligases have not been identified in these processes prior to this identification of MuRF2. In contrast to prior studies where ubiquitination was ligand dependent and resulted in PPAR degradation (as recently reviewed [43]), here we identified that the MuRF2:PPARγ1 ratio determined if degradation occurred (Fig. 6b) and that the physiologically relevant non-degradation of PPARα, PPARβ, and PPARγ1 (Fig. 6a) resulted from multi-mono-ubiquitination of PPAR isoform substrates (Fig. 6e, g).

The ubiquitin ligase:substrate ratio effects on ubiquitination chain type has been studied extensively in cancer with MDM2’s regulation of p53 [86–90]. In these series of elegant studies, investigators identified that low levels of MDM2 induced mono-ubiquitination and nuclear export of p53, whereas high levels of MDM2 promoted poly-ubiquitination and nuclear degradation of p53 [90]. In the context of stress, Li et al. endorsed the notion that non-stressed cells regulated p53 by mono-ubiquitination to circumvent the more extensive investment in energy the poly-ubiquitination and degradation require [89]. Conceptually, this increased energy expenditure may be worthwhile during stress considering that the bigger degradation response leads to apoptosis, whereas mono-ubiquitnation does not [89]. These studies also highlight the highly dynamic process of transcription factor regulation at the post-translational level [87]. Subsequent studies identified that SUMOylation, a process paralleling ubiquitination, can regulate the strength of the MDM2-p53 interaction and participates in the nuclear export [88]. This process appears to involve the stepwise interplay between SUMOylation and ubiquitination of p53 [86]. Very much like p53, all three PPAR isoforms are both SUMOylated and ubiquitinated, so future studies investigating the possible role of MuRF2-regulation of PPAR isoform ubiquitination may take this into consideration. The requirement of SUMOylation for ubiquitination to occur may also explain MuRF2’s apparent regulation of PPARβ activity (Fig. 1a, middle frame) in vivo, but absence of PPARβ ubiquitination in vitro (Fig. 6f) with demonstrable MuRF2 activity but lack of SUMO and/or other interacting proteins.

Another unexpected finding in the present study is the multi-mono-ubiquitination of PPARα and PPARγ1 proteins identified in the in vitro ubiquitination assays (Fig. 6e, g, respectively). In cancer cells, the ubiquitin ligase 14ARF has been reported to di-ubiquitinate p53 in a manner which inhibits MDM2, another 14ARF substrate [91]. Like previous reports of multi-ubiquitinated (e.g. mono- and di-ubiquitination) substrates [92–94], di- or tri-ubiquitination of PPAR does not lead to its degradation in the physiological conditions. Interestingly, 14ARF induces p53-dependent SUMOylation in its target substrates, including MDM2 and NPM, in addition to ubiquitinating the protein [91]. MuRF2’s multi-ubiquitination may offer additional clues into the complex regulation of cardiac PPAR isoforms previously unknown.

We previously identified that MuRF2−/− hearts exhibited alterations in taurine, aspartic acid, and d-malic acid in vivo compared to strain-matched wildtype hearts [37]. In the present study, we expanded these findings in MuRF2−/− hearts after 26 weeks high fat diet to illustrate that differences in taurine, sucrose, glyceric acid, 3-hydroxyflavone, pantothenic acid, and glutamic acid (Fig. 8b). Alternations in taurine and hypotaurine metabolite sets (Fig. 8d) in the MuRF2−/− hearts are interesting given the emerging role of taurine on chronic heart failure. Taurine is an abundant amino acid that influences the heart’s response to stress. It is one of the most abundant amino acids in the left ventricle, acting as an osmoregulator to trigger osmotic preconditioning, a process that activates Akt-dependent cytoprotective signaling [95]. The loss of taurine can depress protein synthesis and reduce energy reserves after cardiac surgery and has been found to be preserved [96]. Specifically, taurine has been shown to attenuate oxidative stress and alleviate heart failure in diabetic rates [97]. Supplementation of taurine in patients with heart failure has been used clinically [98, 99]. Our understanding of cardiac taurine biology is limited, but regulation of taurine by the taurine/Na+ symport is believed to play an important functional role in heart failure and replacement an emerging practice in Japan. It’s role in diabetic cardiomyopathy, in particular, has been found to reduce AGE, oxidized LDL by scavenging malondialdehyde, and hypochlorous acid and downstream HClO-dependent NO reduction [100].

At least three ubiquitin ligases, namely RNF5, TRAF6, and Nedd4 have been described as regulators of autophagy components ATG4B and Beclin1 in non-cardiovascular systems [101]. With the growing appreciation of E3s in regulating autophagy and MuRF2’s interactions with the autophagy-related Nbr1, p62, and LC3 proteins during cardiac myofibril assembly and turnover [41, 102], it was surprising that MuRF2−/− cardiac autophagy was not affected differentially after the high fat diet challenge (Additional file 5: Figure S5b). Despite these provocative parallels, no previous studies of MuRF2’s regulation of autophagic flux have been described, and in the present studies, the lack of endogenous MuRF2 did not affect autophagic flux after high fat diet challenge (Additional file 5: Figure S5b). Similarly, steady state cardiac p62 protein levels were unaffected by the lack of endogenous MuRF2 (Additional file 5: Figure S5b), indicating that cardiac MuRF2 may not have a role in cardiac autophagy or that other MuRF family proteins, such as MuRF1 which has been described in multiple processes [37, 40], are functionally redundant and is compensating in the MuRF2−/− model. As emerging evidence that autophagy plays a role in the pathogenesis of diabetic cardiomyopathy by clearing post-translationally modified proteins, such as advanced glycation end products and the severity of disease [19], targeting autophagy may offer one therapeutic pathway [103]. In the present study, we did not identify that endogenous MuRF2 was protective via this pathway, however.

A host of changes have been described in diabetic cardiomyopathy, characterized by cardiac hypertrophy, inflammation, fibrosis, and apoptosis due to altered insulin signaling and calcium handling [104]. The MuRF family ubiquitin ligases, including MuRF1 and MuRF2 have shown to be critical regulators of cardiomyocyte growth and atrophy. Specifically, both physiological and pathological growth has been attributed to MuRF1 and MuRF2 in the heart [40, 46, 105] and skeletal muscle [106], while MuRF1 regulation of cardiac [106] and skeletal muscle atrophy [107, 108]. While the changes seen in diabetic cardiomyopathy are vast, including alternations in metabolism, structural proteins, signal transduction, and ion channels [109], the crucial role of enhanced PPAR signaling has been central to the pathogenesis of this disease downstream of altered insulin resistance [12, 44]. Regulation of PPAR activity, including post-translational modification-mediated regulation, is a process little understood in any cell type, including the cardiomyocyte. The findings of the current study implicate the first cardiac specific ubiquitin ligase that functionally regulates PPAR isoform signaling, by ubiquitination, inhibiting a central pathway in the pathogenesis of disease. Since the regulation of PPARs are dynamic during the course of diabetic cardiomyopathy [110–113], these studies identify the role of MuRF2 in the pathogenesis of diabetic cardiomyopathy and its regulation of PPAR isoforms, including the post-translational inhibition of PPARγ1 that is cardioprotective in vivo.

## Conclusions

We describe the first mechanism by which an ubiquitin ligase inhibits multiple cardiac PPAR isoforms, to protect against high fat diet-induced diabetic cardiomyopathy. We identified that MuRF2 protein levels increase ~20% during the development of diabetic cardiomyopathy induced by high fat diet. Compared to littermate wildtype hearts, MuRF2−/− hearts exhibit an exaggerated diabetic cardiomyopathy, characterized by an early onset systolic dysfunction, larger left ventricular mass, and higher heart weight. MuRF2−/− hearts had significantly increased PPARα- and PPARγ1-regulated gene expression by RT-qPCR, consistent with MuRF2’s regulation of these transcription factors in vivo. Recent studies have described MuRF2 mutations to contribute to the severity of familial hypertrophic cardiomyopathy. The present study suggests that the lack of MuRF2 activity, as found in these patients, can result in an exaggerated diabetic cardiomyopathy. These present studies also identify MuRF2 as the first ubiquitin ligase to regulate cardiac PPARα and PPARγ1 activities in vivo via post-translational modification without degradation and may represent a novel potential therapeutic target against heart failure in diabetes.

## Additional files

Additional file 1:
**Figure S1.** Analysis of circulating total cholesterol, triglyceride, glucose, insulin, and muscle weight analysis at baseline and after 26 weeks high fat diet challenge. **A.** Fasting blood glucose and fasting serum insulin levels. **B.** Fasting total cholesterol and fasting serum triglyceride levels. **C.** Organ weights at 26 weeks high fat diet of gastrocnemius, soleus, and tibialis anterior. Values represent the mean ± SE (N indicated above bars). Values expressed as Mean ± SE. A one-way ANOVA was performed to determine significance followed by an all pairwise multiple comparison procedure (Holm-Sidak method). #p<0.05, *p<0.001. Additional file 2:
**Figure S2.** Histological analysis of MuRF2-/- mice. **A.** Representative H&E analysis of MuRF2-/- and MuRF2+/+ tissue. **B.** Single MuRF2+/+ heart from mouse found dead 21 weeks high fat diet reveals amorphous way infiltration (arrows) and rare leukocytes infiltrations (*). **C.** Analysis of Masson’s Trichrome stained slides of MuRF2+/+ heart revealed ~3% fibrosis. Additional file 3:
**Figure S3.** mRNA analysis of cardiac PPAR isoform expression in MuRF2 -/- mice. Quantitative RT qPCR analysis of cardiac **A.** PPAR∝ mRNA **B.** PPARβ mRNA and **C.** PPARγ1 mRNA at baseline and 26 weeks after high fat diet compared to sibling-matched wild type hearts. N=5/group. A one-way ANOVA was performed to determine significance followed by an All Pairwise Multiple Comparison Procedure (Holm-Sidak method). *p<0.001. Additional file 4:
**Figure S4.** Detection of cardiac O-GlcNac Protein modifications in MuRF2-/- mice after 26 weeks HFD challenge. **A.** Densitometric analysis of O-GlcNac/βactin immunoblot (**B**). N=3/group. Values expressed as Mean ± SE. A one-way ANOVA was performed to determine significance followed by an All Pairwise Multiple Comparison Procedure (Holm-Sidak method). #p<0.05. Additional file 5:
**Figure S5.** Analysis of MuRF2-/- cardiac NF-κB, IRS-1, cJun signaling and autophagy. **A.** Immunoblot analysis of NF-κB, IRS-1, and cJun reveal no differences in MuRF2-/- and sibling wild type mice (N=3/group). **B.** Analysis of autophagic flux post-Bafilomycin treatment prior to harvest did not detect differences in LC3II isoform, p62, or VPS34 protein levels. Values expressed as Mean ± SE. Statistical analysis was performed using a Student’s t-test. N=3/group. Significance determined as p<0.05. Additional file 6:
**Figure S6.** Pathway analysis of VIP and t-test significant metabolites found in non-targeted metabolomics analysis of MuRF2-/- hearts after high fat diet. N=3/group.

## Abbreviations

c-Jun: jun proto-oncogene
DAG: diacylglycerol
EF%: ejection fraction %
FOXO: forkhead box O
FS%: fractional shortening %
IKK: inhibitor κB kinase
IRS1: insulin receptor substrate-1
JNK: c-JUN NH 2-terminal kinase
LV: left ventricular
LV Vol: left ventricular volume
LVESD: left ventricular end systolic dimension
LVEDD: left ventricular end diastolic dimension
MDM2: mouse double minute 2 homolog (ubiquitin ligase)
MuRF2: muscle ring finger-2
NFκB: nuclear factor kappa B
O-GlcNAc: *O*-linked *N*-acetylglucosamine
p53: aka TP53-tumor protein 53
PPAR: peroxisome proliferator activating receptor
PCA: principal components analysis
PLS-DA: partial least squares discriminant analysis
PPRE: PPAR-response element
PKC: protein kinase C
SRF: serum response factor
VIP: variable interdependent parameters
