# Supplementary material for: MuRF2 regulates PPARγ1 activity to protect against diabetic cardiomyopathy and enhance weight gain induced by a high fat diet
Source: Cardiovasc Diabetol. 2015 Aug 5;14:97. doi: 10.1186/s12933-015-0252-x (PMC4526192; doi:10.1186/s12933-015-0252-x)
Supplement: Additional file 1: — Figure S1. Analysis of circulating total cholesterol, triglyceride, glucose, insulin, and muscle weight analysis at baseline and after 26 weeks high fat diet challenge. A. Fasting blood glucose and fasting serum insulin levels. B. Fasting total cholesterol and fasting serum triglyceride levels. C. Organ weights at 26 weeks high fat diet of gastrocnemius, soleus, and tibialis anterior. Values represent the mean ± SE (N indicated above bars). Values expressed as Mean ± SE. A one-way ANOVA was performed to determine significance followed by an all pairwise multiple comparison procedure (Holm-Sidak method). #p<0.05, *p<0.001. [file 12933_2015_252_MOESM1_ESM.pdf]

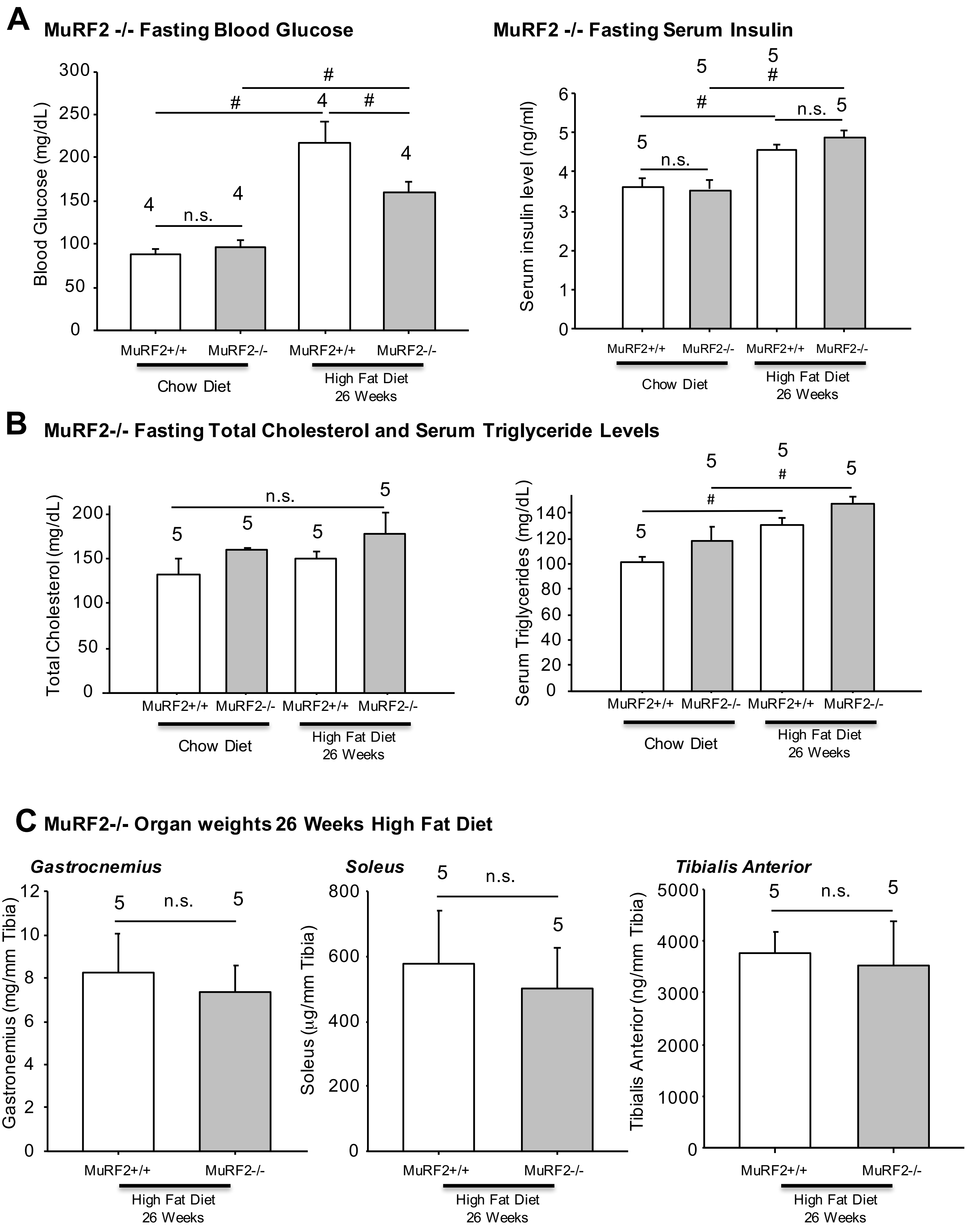
A
MuRF2 -/- Fasting Blood Glucose
Blood Glucose (mg/dL)
300
250
200
150
100
50
0
#
#
4
#
4
4
n.s.
4
MuRF2+/+
MuRF2-/-
MuRF2+/+
MuRF2-/-
Chow Diet
High Fat Diet
26 Weeks
MuRF2 -/- Fasting Serum Insulin
Serum insulin level (ng/ml)
6
5
4
3
2
1
0
5
5
#
#
n.s.
5
5
n.s.
MuRF2+/+
MuRF2-/-
MuRF2+/+
MuRF2-/-
Chow Diet
High Fat Diet
26 Weeks
B
MuRF2-/- Fasting Total Cholesterol and Serum Triglyceride Levels
Total Cholesterol (mg/dL)
200
150
100
50
0
n.s.
5
5
5
5
MuRF2+/+
MuRF2-/-
MuRF2+/+
MuRF2-/-
Chow Diet
High Fat Diet
26 Weeks
Serum Triglycerides (mg/dL)
140
120
100
80
60
40
20
0
5
5
#
5
#
5
MuRF2+/+
MuRF2-/-
MuRF2+/+
MuRF2-/-
Chow Diet
High Fat Diet
26 Weeks
C
MuRF2-/- Organ weights 26 Weeks High Fat Diet
Gastrocnemius
Gastronemius (mg/mm Tibia)
12
10
8
6
4
2
0
5
n.s.
5
MuRF2+/+
MuRF2-/-
High Fat Diet
26 Weeks
Soleus
Soleus (μg/mm Tibia)
800
600
400
200
0
5
n.s.
5
MuRF2+/+
MuRF2-/-
High Fat Diet
26 Weeks
Tibialis Anterior
Tibialis Anterior (ng/mm Tibia)
5000
4000
3000
2000
1000
0
5
n.s.
5
MuRF2+/+
MuRF2-/-
High Fat Diet
26 Weeks

Figure S1.
