# Supplementary figures and images for: MuRF2 regulates PPARγ1 activity to protect against diabetic cardiomyopathy and enhance weight gain induced by a high fat diet
Source: Cardiovasc Diabetol. 2015 Aug 5;14:97. doi: 10.1186/s12933-015-0252-x (PMC4526192; doi:10.1186/s12933-015-0252-x)

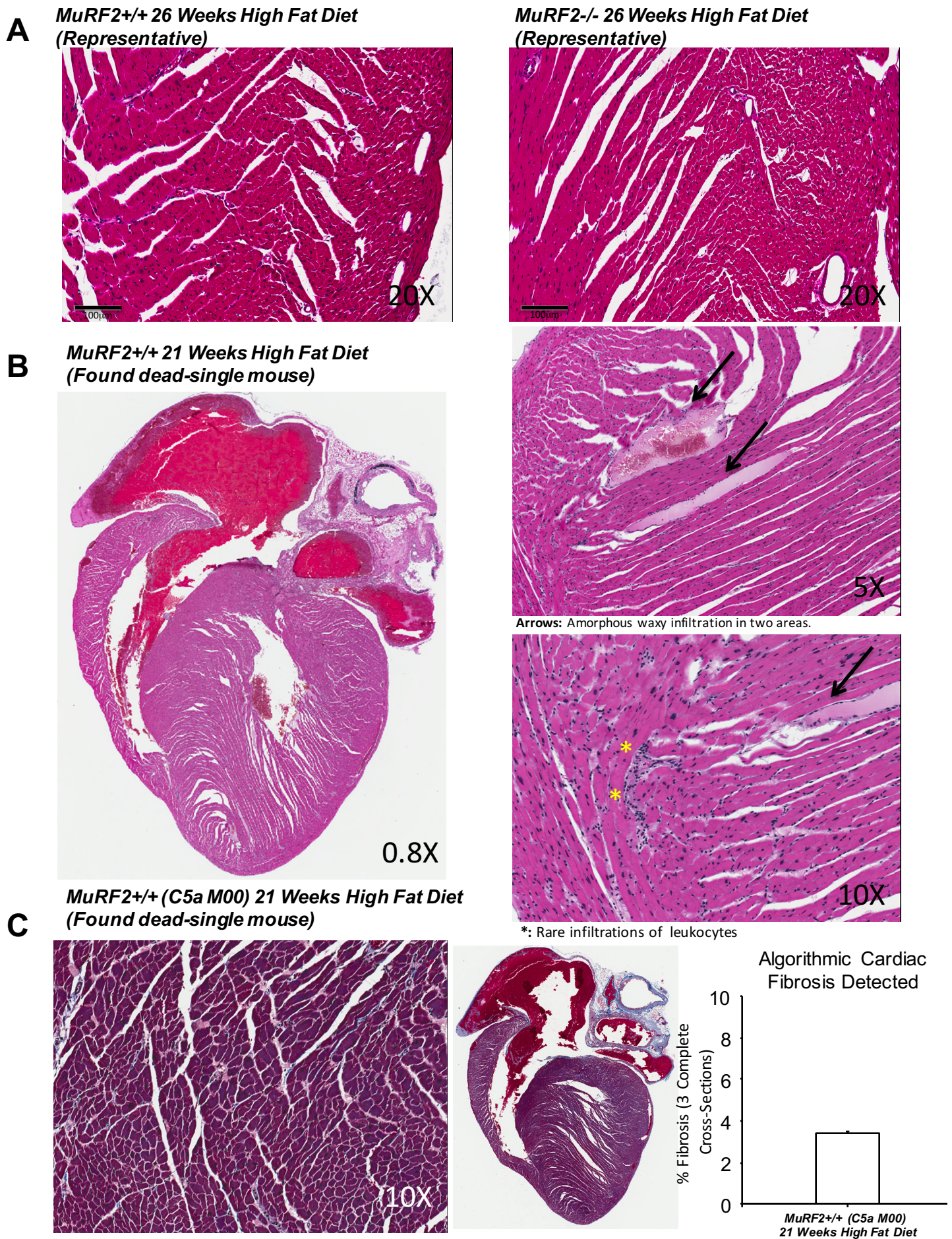

**Figure S2.**

Supplement: Additional file 2: — Figure S2. Histological analysis of MuRF2-/- mice. A. Representative H&E analysis of MuRF2-/- and MuRF2+/+ tissue. B. Single MuRF2+/+ heart from mouse found dead 21 weeks high fat diet reveals amorphous way infiltration (arrows) and rare leukocytes infiltrations (*). C. Analysis of Masson’s Trichrome stained slides of MuRF2+/+ heart revealed ~3% fibrosis. [file 12933_2015_252_MOESM2_ESM.pdf]

# A

## PPAR $\alpha$

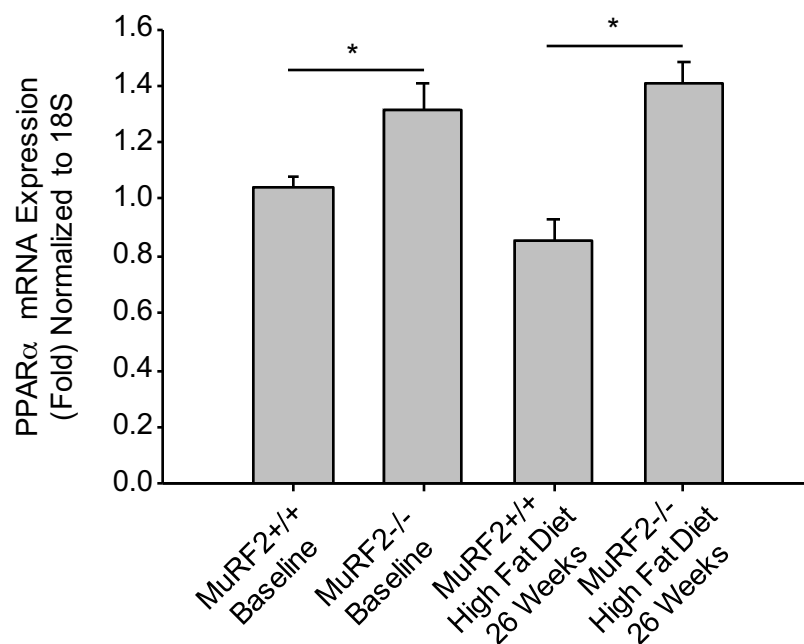

# B

## PPAR $\beta$

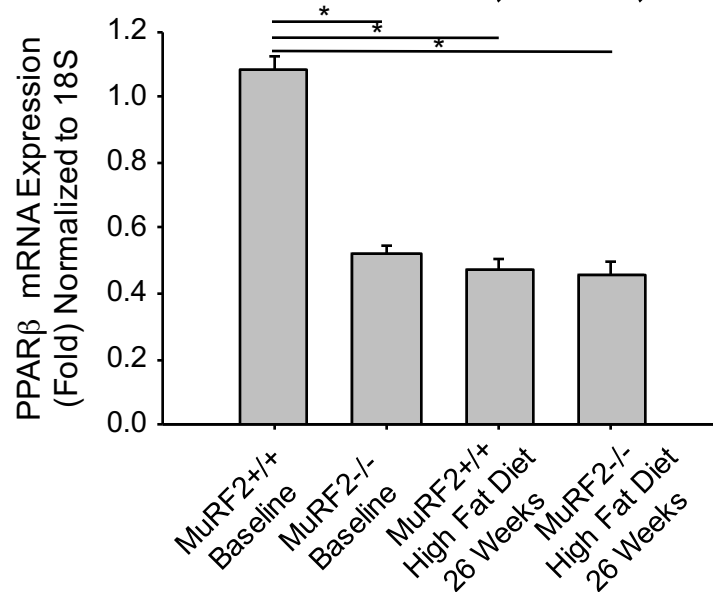

# C

## PPAR $\gamma$ 1

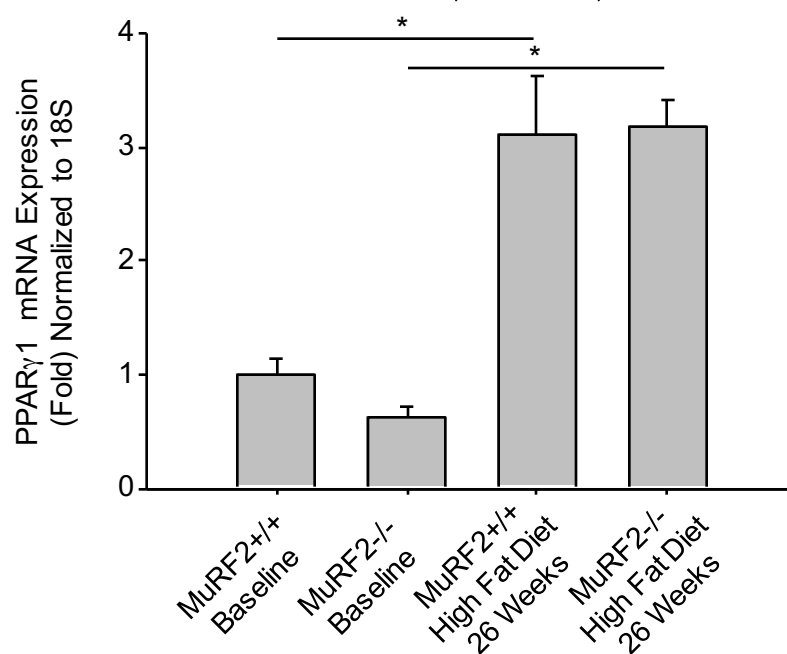

Figure S3.

Supplement: Additional file 3: — Figure S3. mRNA analysis of cardiac PPAR isoform expression in MuRF2 -/- mice. Quantitative RT qPCR analysis of cardiac A. PPAR∝ mRNA B. PPARβ mRNA and C. PPARγ1 mRNA at baseline and 26 weeks after high fat diet compared to sibling-matched wild type hearts. N=5/group. A one-way ANOVA was performed to determine significance followed by an All Pairwise Multiple Comparison Procedure (Holm-Sidak method). *p<0.001. [file 12933_2015_252_MOESM3_ESM.pdf]

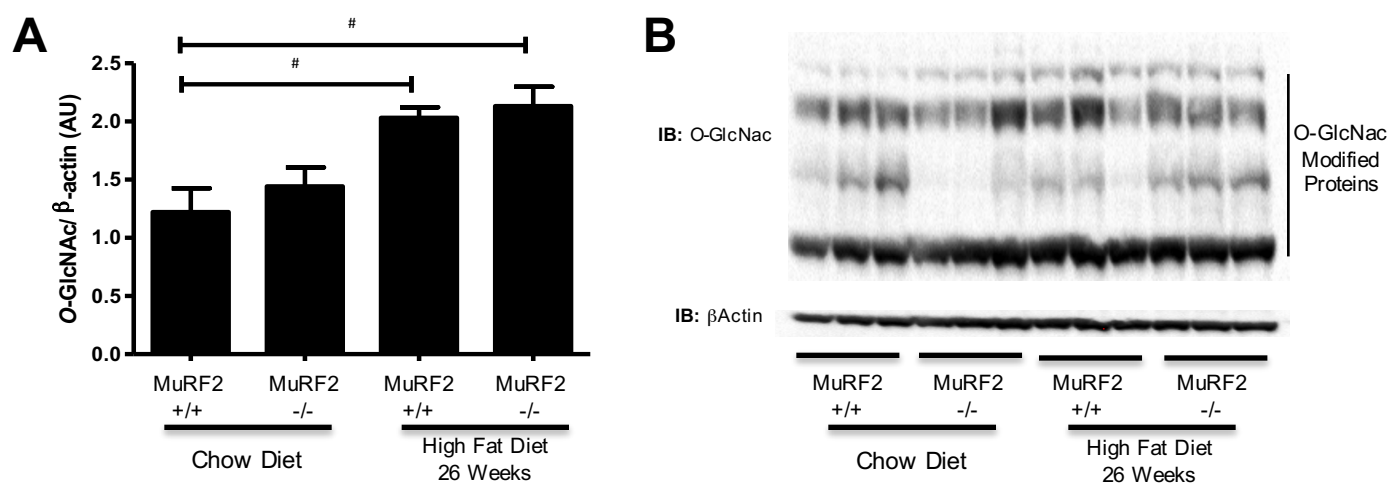

**Figure S4.**

Supplement: Additional file 4: — Figure S4. Detection of cardiac O-GlcNac Protein modifications in MuRF2-/- mice after 26 weeks HFD challenge. A. Densitometric analysis of O-GlcNac/βactin immunoblot (B). N=3/group. Values expressed as Mean ± SE. A one-way ANOVA was performed to determine significance followed by an All Pairwise Multiple Comparison Procedure (Holm-Sidak method). #p<0.05. [file 12933_2015_252_MOESM4_ESM.pdf]

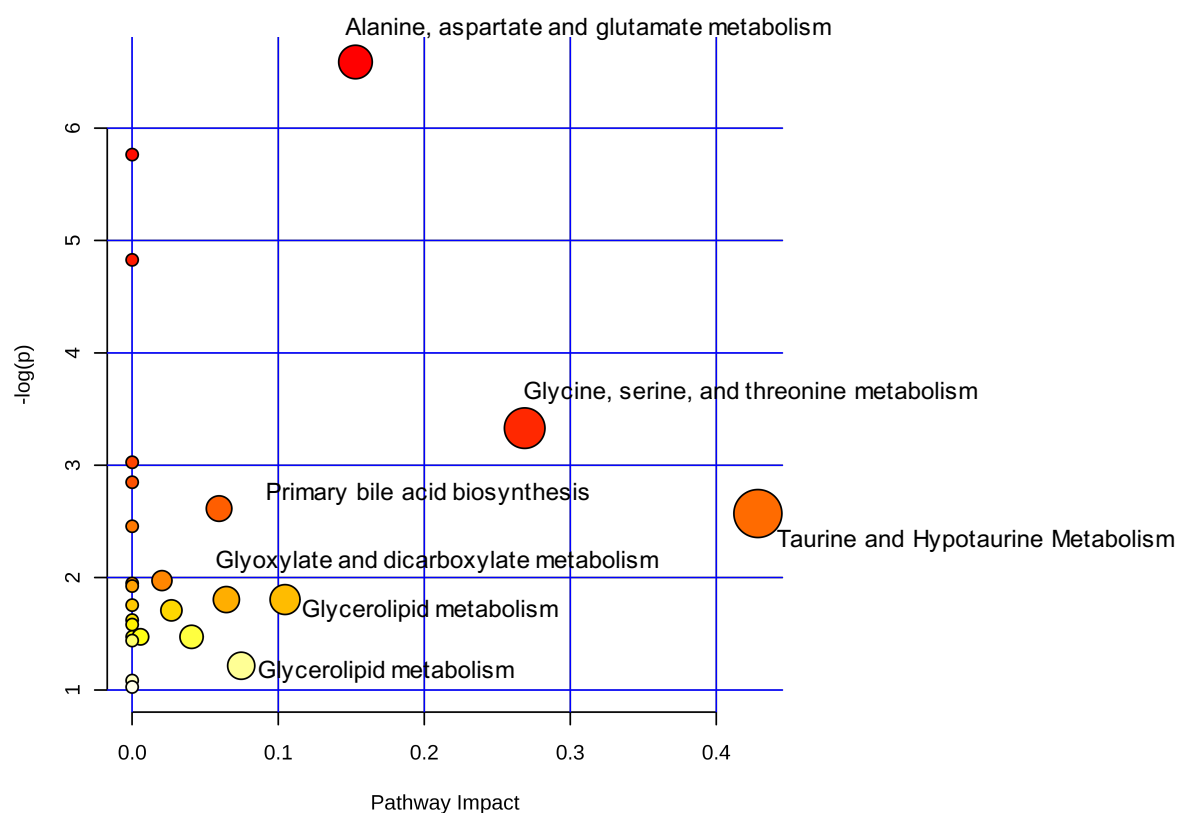

Figure S6.

Supplement: Additional file 6: — Figure S6. Pathway analysis of VIP and t-test significant metabolites found in non-targeted metabolomics analysis of MuRF2-/- hearts after high fat diet. N=3/group. [file 12933_2015_252_MOESM6_ESM.pdf]
